# An Overview of Reviews on Interprofessional Collaboration in Primary Care: Barriers and Facilitators

**DOI:** 10.5334/ijic.5589

**Published:** 2021-06-22

**Authors:** Cloe Rawlinson, Tania Carron, Christine Cohidon, Chantal Arditi, Quan Nha Hong, Pierre Pluye, Isabelle Peytremann-Bridevaux, Ingrid Gilles

**Affiliations:** 1 Center for Primary Care and Public Health (Unisanté), Route de Corniche 10, 1010 Lausanne, Switzerland; 2 Department of Family Medicine, McGill University, 5858, Chemin de la Côte-des-Neiges, Montreal, Quebec, Canada

**Keywords:** interprofessional, collaboration, primary care, barriers, facilitators, overview, review

## Abstract

**Introduction:**

Interprofessional collaboration (IPC) is becoming more widespread in primary care due to the increasing complex needs of patients. However, its implementation can be challenging. We aimed to identify barriers and facilitators of IPC in primary care settings.

**Methods:**

An overview of reviews was carried out. Nine databases were searched, and two independent reviewers took part in review selection, data extraction and quality assessment. A thematic synthesis was carried out to highlight the main barriers and facilitators, according to the type of IPC and their level of intervention (system, organizational, inter-individual and individual).

**Results:**

Twenty-nine reviews were included, classified according to six types of IPC: IPC in primary care (large scope) (n = 11), primary care physician (PCP)-nurse in primary care (n = 2), PCP-specialty care provider (n = 3), PCP-pharmacist (n = 2), PCP-mental health care provider (n = 6), and intersectoral collaboration (n = 5). Most barriers and facilitators were reported at the organizational and inter-individual levels. Main barriers referred to lack of time and training, lack of clear roles, fears relating to professional identity and poor communication. Principal facilitators included tools to improve communication, co-location and recognition of other professionals’ skills and contribution.

**Conclusions:**

The range of barriers and facilitators highlighted in this overview goes beyond specific local contexts and can prove useful for the development of tools or guidelines for successful implementation of IPC in primary care.

## Introduction

The ageing population and growing burden of chronic diseases have brought new challenges for healthcare systems, and particularly primary care, with higher risks of care fragmentation, poorer quality of care, and higher health costs. This has led to the development of new models of care, such as those based on interprofessional collaboration (IPC) to improve health care processes, patient outcomes and reduce health costs in primary care [1,2,3,4]. IPC in primary care can be defined as an integrative cooperation of different healthcare professionals, blending complementary competences and skills, making possible the best use of resources [3]. Several studies have shown positive effects of working as a team, including better care continuity and coordination, beneficial changes in patient behavior, improvement of patient symptoms and satisfaction through better response to their needs [5,6,7,8,9,10]. However, studies also suggest that its implementation can be challenging [11,12]. In practice, IPC can be compromised when professionals are not convinced of its benefits for patients, or when primary care providers perceive it as a loss of continuous and holistic patient care, a loss of professional identity [13] or of their jobs’ attributes [14]. The lack of knowledge of other professionals’ skills, reluctance to change [11,15], and the absence of interprofessional education in curriculums [12,16,17] have also been reported as hindering practice of IPC. Currently, the growing interest in IPC in primary care, reflected in the amount of published literature, including several systematic reviews, suggests that it is crucial to obtain a comprehensive view about what hinders and facilitates the practice of IPC. We thus conducted an overview of reviews (i.e. review of systematic reviews) of IPC in the primary care setting, to analyze and synthesize results from existing reviews, in terms of effectiveness, barriers and facilitators, and theoretical models or conceptual frameworks. The current article presents the results related to the identification of the main barriers and facilitators of IPC (also referred to as factors) in primary care.

## Methods

Overview of reviews, also known as umbrella review, meta-review or review of reviews, aims to integrate information from multiple systematic reviews, by using a rigorous methodological process, to offer a comprehensive synthesis regarding a specific subject by adopting a broader scope than is proposed in each systematic review [18,19,20]. We performed the overview of reviews in alignment with the Preferred Reporting Items for Systematic Reviews and Meta-Analysis (PRISMA) statement [21] and in accordance with the recommendations outlined by the Joanna Briggs Institute [18]. Review eligibility criteria, methods of review identification and selection, as well as methods of data extraction and synthesis were pre-defined in a protocol registered on PROSPERO (CRD42017069922).

### Eligibility criteria

Eligibility criteria covered three domains. First, reviews had to be centered on IPC, defined as an ongoing partnership and/or interaction between at least two healthcare professionals from different backgrounds working together to improve patients’ care. More specifically, two forms of collaboration were considered: 1) collaboration within primary care practices/institutions and 2) collaboration between primary care provider(s) (primary care physician(s) (PCP) or primary care nurse(s), such as for example family physicians/practitioners, general physicians/practitioners, nurse practitioners, practice nurses) and healthcare professional(s) working outside the primary care setting. We excluded reviews focusing on interprofessional education, on a specific aspect of IPC, on instruments measuring IPC or reviews primarily targeting structural collaboration and not involving interactions between healthcare providers. Second, the setting of the review had to be primary care, as defined by Starfield [22], the Institute of Medicine [23] or the World Health Organization [24]. The IPC intervention had to include at least one primary care provider if the setting was not clearly mentioned. Third, we only included reviews that had been conducted in a systematic manner [25], i.e. with a rigorous and explicit methodology for the search strategy, study selection, quality appraisal, and synthesis of results. Reviews including only qualitative, only quantitative (with or without meta-analysis), or a combination of qualitative, quantitative or mixed methods studies, as well as conceptual and theoretical work were eligible for inclusion.

### Search strategy

The search strategy, elaborated with a librarian, included MeSH terms and words relating to the concepts of IPC, primary care and review (S1 Table). The search was carried out on May 10^th^, 2017 in nine databases: MEDLINE, EMBASE, CINAHL, PsycINFO, the Cochrane Database of Systematic Reviews, the Database of Abstract Reviews of Effects (DARE), JBI Database of Systematic Reviews and Implementation Reports, PROSPERO, and Epistemonikos. The search was updated on January 31^st^, 2019. We checked reference lists of included reviews for additional reviews.

### Study selection and data extraction

Screening of titles and abstracts (stage 1), and of full-text papers (stage 2) were carried out in the Covidence platform. A standardized predefined data extraction form (S2 Table) was used to extract data from eligible reviews. We contacted corresponding authors for missing or incomplete data. Results reported in the reviews were extracted separately according to whether they were presented as barriers or facilitators of IPC. Three authors took part in these processes (T.C., C.R. and C.A.). Among them, two independent reviewers selected all articles (using title/abstract then full-text) and extracted data. Disagreements were resolved during discussions between authors. When necessary, a fourth author was consulted for final decision (I.P.B.).

### Quality assessment

The methodological quality of each included review was assessed by two independent reviewers (among T.C., C.R., or C.A.) using the ROBIS tool [26]. We attributed a *Low, High* or *Unclear* risk of bias to each domain (study eligibility criteria, identification and selection of studies, data collection and study appraisal, and synthesis and findings), and to the review as a whole. These results served as an indicative purpose to inform on the quality of the included reviews. No reviews were eliminated according to their risk of bias.

### Degree of overlap

To avoid interpretation biases and address the inclusion of primary studies in more than one review [27], we calculated the degree of overlap by using the “Corrected Covered Area” (CCA) measure [28]; a CCA value ≤5% being considered as a slight overlap, and values ≥15% as a very high overlap [28].

### Synthesis of results

The textual content relating to barriers and facilitators identified in the reviews were coded separately using thematic synthesis according to the Braun & Clarke method [29] and using the Maxqda® (v.11) software. Themes were progressively generated by following an inductive approach, to provide broad types of barriers and facilitators. The latter were then classified into four levels: system (determinants from the environment outside the organization), organizational (conditions within the organization), inter-individual (relating to the interpersonal relationship between professionals and/or within the team), and individual (specific to the individual). Moreover, since the included reviews targeted specific types of collaboration, in terms of setting and professionals involved, we classified them in broader types of IPC. Then, we tagged the coded segments according to this classification in order to identify barriers and facilitators that were both the most reported and the most specific for each type of IPC. We chose to present barriers and facilitators by type of collaboration in the form of tables and narratively.

## Results

### Search results

**Figure 1:**
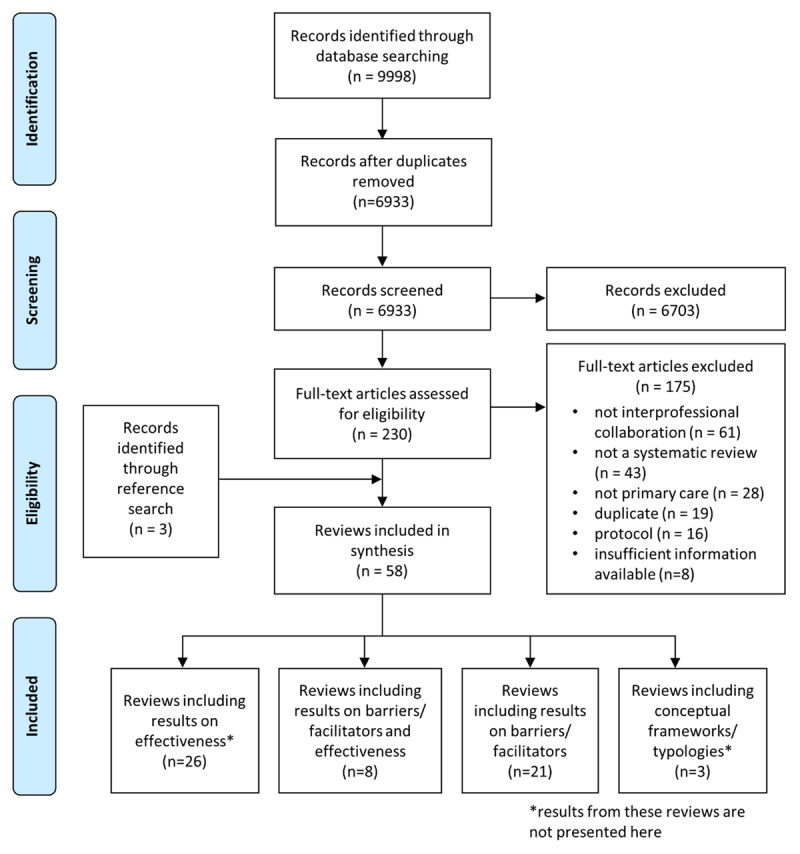
Flow chart.

From 9998 records identified, 230 full text articles were screened. Of the 230 screened full text articles, there was disagreement on 24 (10.4%). Thirteen of these disagreements were solved in discussion between the three authors involved in the screening (T.C., C.R. and C.A.). For the 11 remaining, disagreements were solved by consulting a fourth author (I.P.B.). Fifty-eight reviews met the selection criteria and were included in this overview, of which 29 reported factors hindering or facilitating IPC (***Figure 1***). This corresponded to 1,091 primary studies (9 to 251 primary studies per review). The CCA value was of 0.6%, indicating a slight degree of overlap.

### Characteristics of included reviews

**Figure 2:**
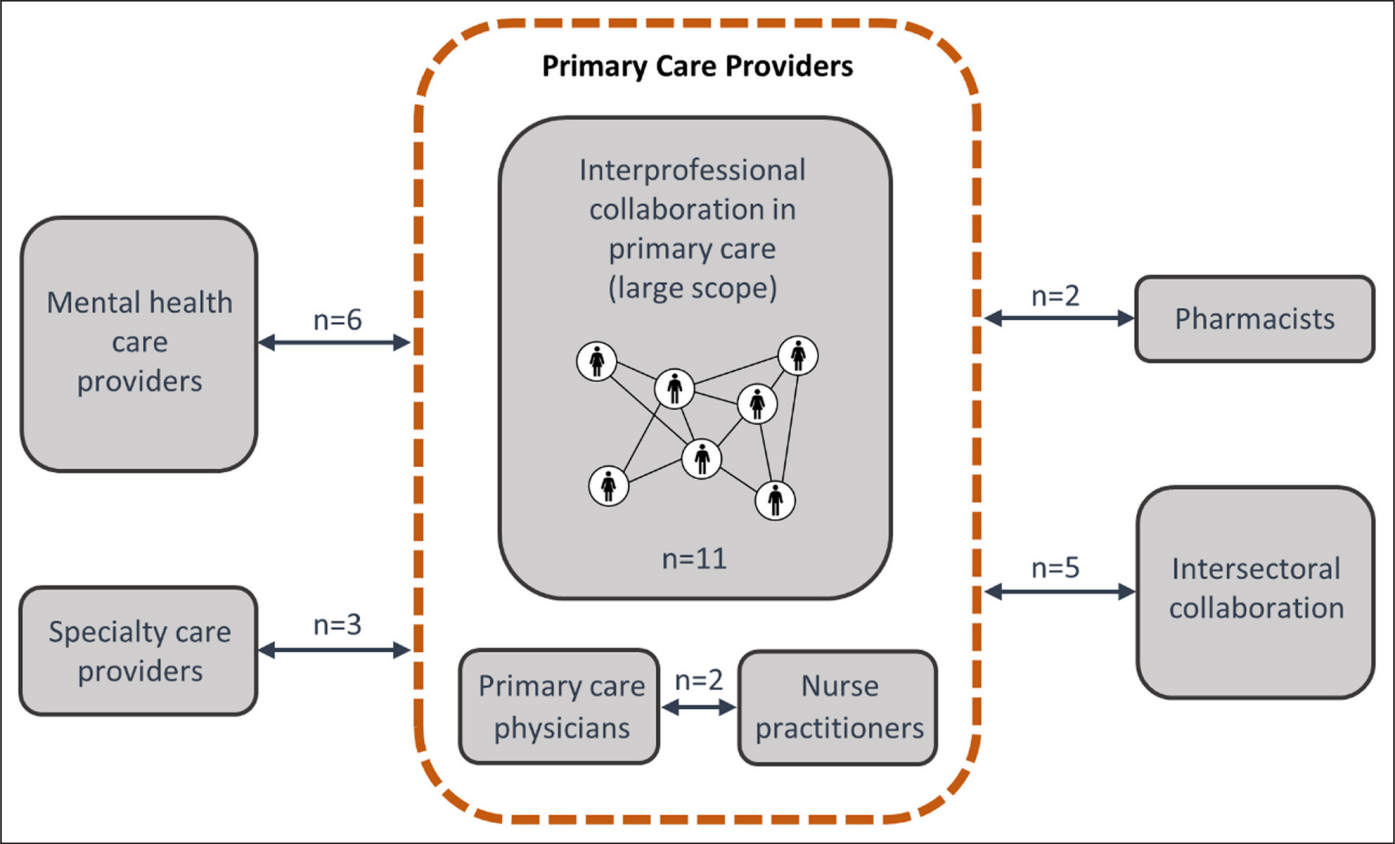
Six types of interprofessional collaboration identified.

Of the 29 reviews, 20 were mixed methods reviews, six were qualitative, and three were quantitative. The most frequently used method of synthesis was thematic synthesis (n = 16), followed by narrative synthesis (n = 8), framework synthesis (n = 2), taxonomic analysis (n = 1), realist approach (n = 1) and pragmatic meta-aggregative approach (n = 1). We identified six types of IPC, based on the authors’ way of defining the setting and the type of health professionals involved in IPC (***Figure 2***): (1) *IPC in primary care (large scope)* included reviews focusing on healthcare professionals working in interprofessional primary care teams, without targeting specific professions (n = 11); (2) *PCP-nurse collaboration* corresponded to reviews focusing on collaboration between physicians and nurses in primary care, for example in general practices or reporting nurse practitioners’ views and experiences (n = 2); (3) *PCP-specialty care provider collaboration* included reviews targeting collaboration between a PCP and a specialist (e.g., palliative care providers, oncologists, psychiatrists, cardiologists) (n = 3); (4) *PCP-pharmacist collaboration* corresponded to reviews specifically addressing collaboration between PCP and community pharmacists (n = 2); (5) *PCP-mental health care provider collaboration* contained reviews reporting interventions implemented in primary mental health settings, such as “Collaborative care” models (multi-professional approaches to patient care typically involving a PCP, a mental health specialist, and a case manager) (n = 6). The final type of IPC, (6) *intersectoral collaboration*, included reviews on the collaboration between primary care and other sectors (nursing homes, sport sector, oral health or public health) (n = 5). The risk of bias was low for 11 reviews, high for 15 and unclear for 3. Characteristics of the reviews are presented in S3 Table.

**Figure 3:**
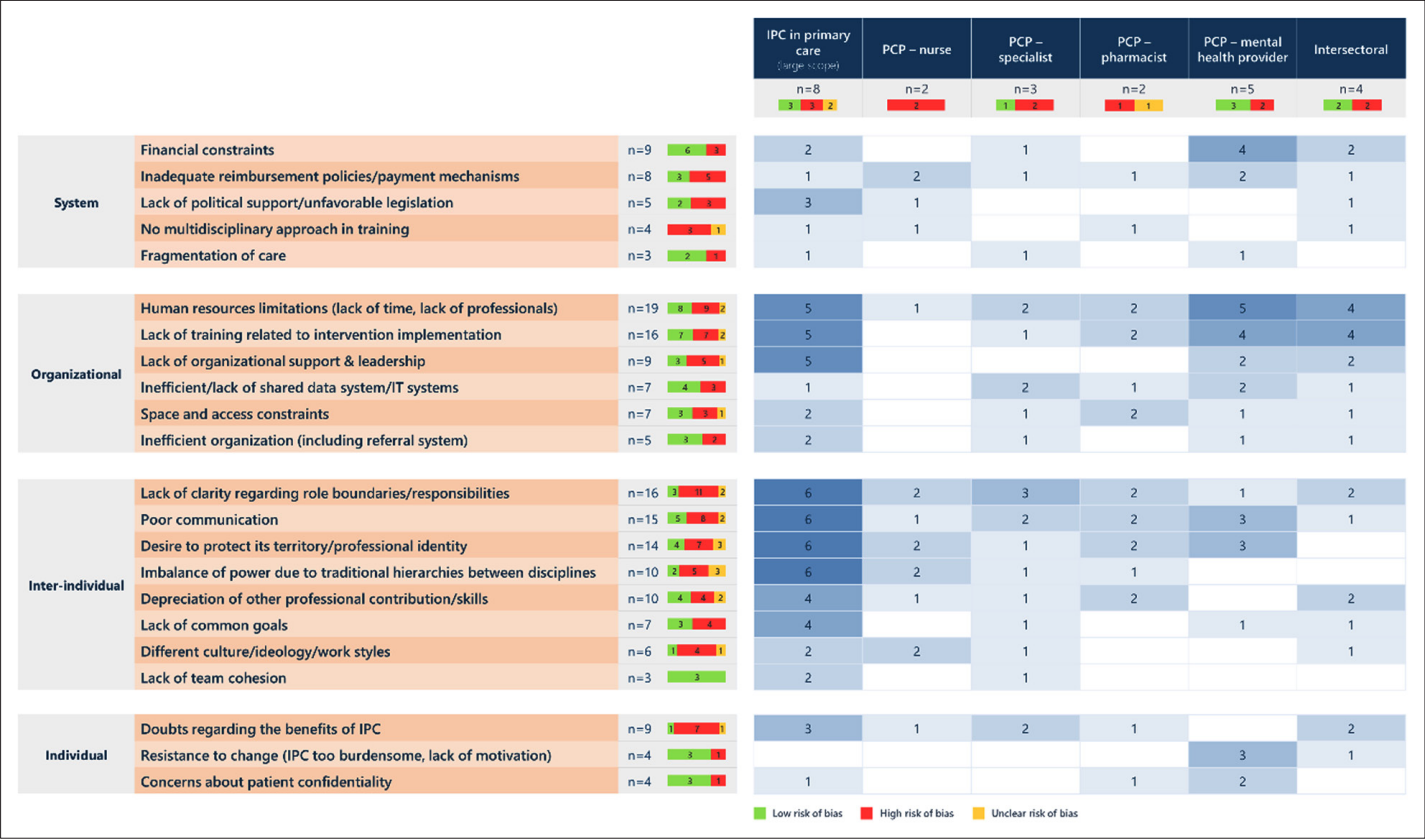
Barriers to interprofessional collaboration with the number of reviews reporting them, by type of collaboration.

**Figure 4:**
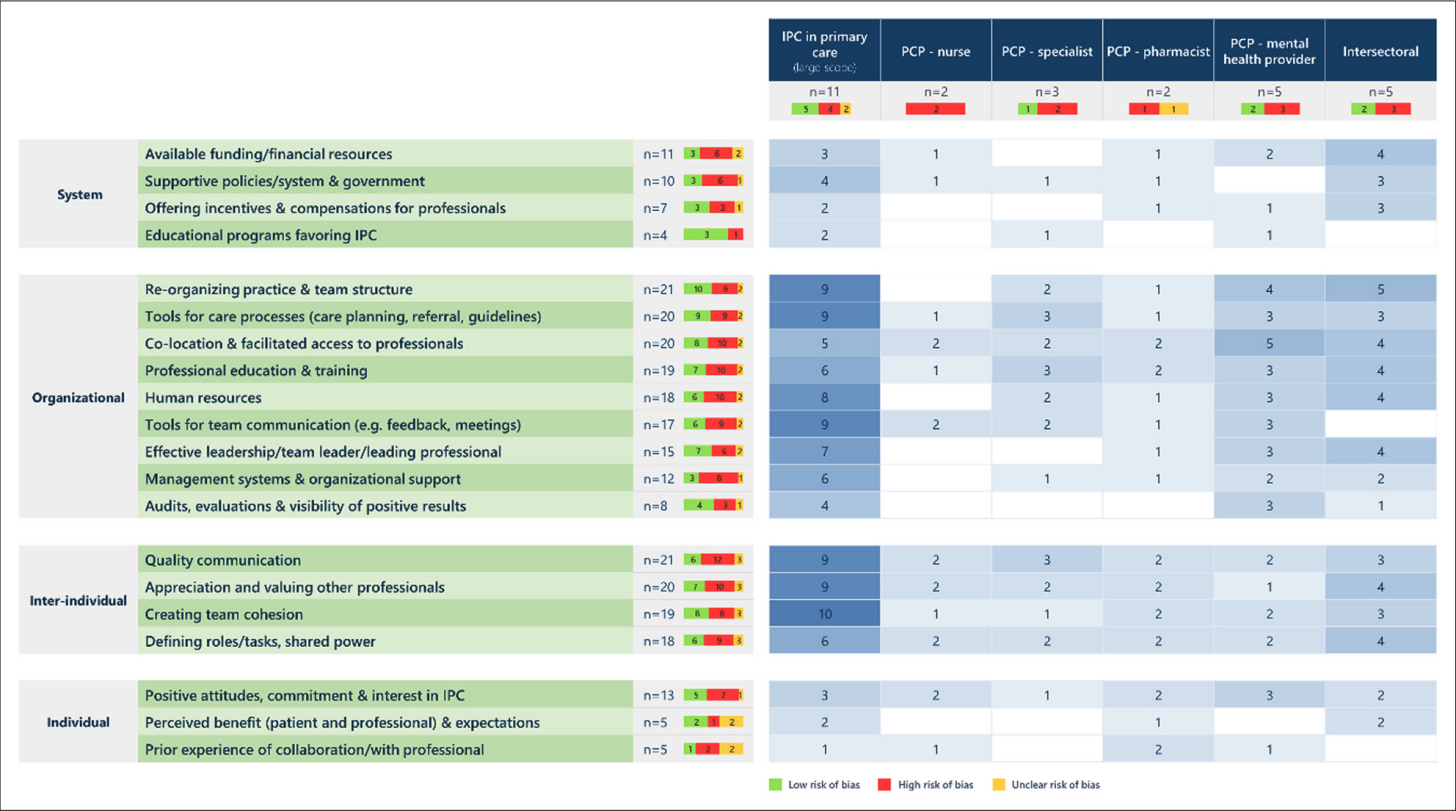
Facilitators of interprofessional collaboration with the number of reviews reporting them, by type of collaboration.

The synthesis identified 22 types of barriers and 20 types of facilitators impacting IPC across all types of collaboration (***Figures 3*** and ***4***). Among the reported barriers, four were common to all types of IPC, 16 were reported in three or more types of collaboration and two were specific to one or two types only. Regarding facilitators, eight were common to all types of IPC, 12 were reported in three or more types of collaboration and none were specific to one or two types of IPC. In the next sections, for each of the six types of IPC, we present first the findings regarding barriers and then regarding facilitators (S4 Table for detailed results).

### Barriers and facilitators of interprofessional collaboration

#### IPC in primary care (large scope)

At the system level, the barriers that were most often reported concerned financial barriers (e.g., lack of long-term funding, inadequate reimbursement policies) [11,30], followed by the lack of leadership at national/political level and the lack of support in legal constraints for the expansion of roles [11,30,31]. At the organizational level, limitations in human resources (e.g., lack of time and skilled professionals) led to an increased workload [11,15,31,32,33]. The lack of professionals’ training in IPC implementation and of organizational support in this process were also mentioned [11,15,31,32,34,35]. At the inter-individual level, the imbalance of power between professionals, due to hierarchies between disciplines (especially physicians versus other professionals) at a structural level [11,15,32,33,34,35] was mentioned. The lack of clarity regarding functions and scopes of other professionals and fear of loss of territory/professional identity in newly defined roles [11,15,31,32,33,34,35] was associated with the depreciation of other professionals’ contributions and skills as well as the lack of common vision and goals [11,15,31,32,35]. Poor or deficient communication between actors was also an important barrier [11,15,31,32,33,35]. At the individual level, professionals were concerned about the benefits of collaboration for their patients [15,34,35].

Facilitators at the organizational and inter-individual levels were particularly frequently cited in this type of collaboration. At the organizational level, reinforcement of human resources, with an equitable involvement of professionals and available time [11,15,32,33,34,35,36,37], re-organization of practice and, more specifically, team composition with formalized partnerships and coordination rules were often identified [11,15,30,32,33,34,36,37,38]. The use of tools to improve care processes (e.g., care planning, referral, guidelines) was also mentioned [11,15,30,32,33,34,36,37,38]. Organizing regular meetings and feedback, using clear communication routines or information channels were also facilitators of IPC [15,30,31,32,33,34,35,36,37,38]. Supportive institutions and having a team leader or champion to organize interprofessional collaboration was also mentioned [11,30,31,32,33,34,35,36,37,38]. At the inter-individual level, effective, openly shared knowledge and information regarding patients and moments of informal face-to-face discussions [15,30,31,32,33,34,35,36,37,38] were important. Valorization of other professionals’ work and understanding of their roles, trust and respect between professionals [11,15,32,33,34,35,36,37,38], shared interests, goals and a common vision, and creation of team cohesion through team building were also identified [11,15,32,33,34,35,36,37,38].

#### PCP-nurse collaboration

At the system level, inadequate reimbursement policies and problems with payment mechanisms for nurses’ services (poor reimbursement or insufficient financial support) were the most frequently reported barriers [45,46]. Besides these legal and financial issues, barriers were mostly reported at the inter-individual level where traditional hierarchies between both disciplines and ideological differences in practice and cultural perception of care (biomedical versus experiential) led to power struggles and difficulties regarding professional identity. In fact, whereas nurses feared disadvantages due to their extended roles in the absence of clear definitions of roles boundaries, physicians misunderstood these extended roles.

Facilitators at the organizational level included tools for team communication (e.g., regular meetings, open channels of communication, use of technologies) and close physical proximity between professionals [45,46]. At the inter-individual level, definition of roles and responsibilities, the acceptation of other professionals’ views, competences and practices, and shared leadership were reported [45,46]. At the individual level, a positive attitude and interest in IPC was identified [45,46].

#### PCP-specialty care provider collaboration

Barriers at the organizational level concerned lack of time, work overload [47,48] and the lack of adequate electronic data sharing solutions [47,49]. The lack of PCP experience and uncertainty in knowledge also limited patients’ follow-up [49]. At the inter-individual level, discrepancies in role definitions between primary care physicians and specialty care providers, and lack of clarity in role boundaries led some specialists to assume a PCP role [47,48,49]. Lack of and poor communication also weakened IPC [47,49]. At the individual level, doubts from specialty care providers regarding IPC benefits limited patients’ referrals to primary care physicians during acute phases of disease [48,49].

Facilitators were mainly at the organizational level. These included providing tools for care processes, especially shared care guidelines or pathways, favoring patient data transmission (electronic medical records, survivorship care plans), and improving PCP knowledge and experience in specialty care [47,48,49]. Reorganizing practice [47,49], favoring proximity and access between professionals [48,49], increasing resources in trained staff and personnel, and providing tools for communication [48,49] were also mentioned. At the inter-individual level, effective communication and information exchange (timely, relevant detail) [47,48,49], and agreement on role definition and sharing of decision-making [47,48] facilitated IPC.

#### PCP-pharmacist collaboration

Barriers at the organizational level included lack of available time and specific training on IPC, difficulty for pharmacists to access PCPs and not working in geographically close areas [50,51]. At the inter-individual level, lack of clear role boundaries and responsibilities, especially the lack of knowledge about the other profession were reported. These were associated with the fear of a weakened professional identity, and a lack of or deficient communication [50,51]. Moreover, depreciation of pharmacists by PCPs was also an important barrier, especially regarding confidence in pharmacists’ skills and experience in patient care and perception of pharmacists as retailers, which led to lack of respect and trust [50,51].

At the organizational level, facilitators concerned access to professionals through the use of integrated settings or co-located spaces, increasing proximity between PCPs and pharmacists, and joint training [50,51]. At the inter-individual level, facilitators focused on establishing a respectful environment in which professionals’ skills would be valued, with mutual recognition, respect and trust, in addition to clearly defining responsibilities [50,51]. At the individual level, prior experience of IPC or informal collaboration during formative years reinforced willingness to engage in IPC initiatives [50,51].

#### PCP-mental healthcare provider collaboration

Barriers at the system level concerned financial constraints in general (absence of long-term funding solutions) [39,40,41,42], inadequate reimbursement policies and problems with payment mechanisms such as compensations and reimbursements [39–41]. At the organizational level, barriers concerned the limited number of skilled professionals involved, of time available, and work overload [39,40,41,42,43]. In addition, the lack of specific training on IPC led to professionals’ unfamiliarity with the IPC model and difficulty for non-mental healthcare providers in managing patients with mental health issues [39,40,41,42]. At the inter-individual level, lack of communication between professionals and the threat to professional identity (mainly for PCP) [41,42,43] were reported. Barriers at the individual level included professional’s resistance to change and the perception of IPC as burdensome and resource-consuming to implement [39,40,42]. Professionals were also concerned about patient confidentiality through the use of shared medical records [39,41].

Facilitators concerned mainly the organizational level and included the importance of proximity between professionals to facilitate access to each other (through co-location, full-time presence) [40,41,42,43,44], reorganizing practice, and including a case manager to the collaboration [41–44]. The implementation of tools such as standardized care pathways, scheduled follow-ups and structured management plans to improve care processes, as well as regular meetings, and systematic feedback to improve communication [41,42,43] were also mentioned. Effective leadership by a physician champion, and visibility of the benefits of IPC through audits and evaluations were also mentioned [41,42,43]. At an individual level, a strong engagement of professionals was reported as facilitating IPC [41,42,43].

#### Intersectoral collaboration

Barriers were characterized at the system level by financial constraints, including uncertain or unstable funding and costly IPC implementation, lack of political support due to low prioritization, and insurance specificities (i.e. separation of medical and dental treatment in insurance systems) [52]. At the organizational level, available time and professionals’ retention in programs [52,53,54,55], insufficient training and lack of skills [52,53,54,55] were mentioned. Lack of PCP engagement in leadership due to poor incentives or absent administrative infrastructure to facilitate cross-domain operability were also reported [54,55]. At the inter-individual level, depreciation of others’ contribution and lack of time to install a trusting environment between professionals [53,55] hindered IPC. Finally, doubts about IPC benefits [52,54] were identified at the individual level.

Facilitators at the system level concerned funding, including its stabilization through strong government, stakeholders or non-profit organizations’ support, introduction of incentives for team involvement and compensation of time used for IPC activities [52,54,55,56]. Developing a common vision between different partners such as authorities and communities, and political willingness to support IPC [52,53,56] were also reported. At the organizational level, reorganizing practices by motivating professionals to work in a multi-disciplinary approach, adopting a flexible community driven definition of care, formalizing coordination and dedicating time to IPC [52,53,54,55,56] were important facilitators. Improving professionals’ training and involving the whole staff by using a “bottom-up” approach were also described as crucial [52,53,54,55,56]. A strong engagement of the team supported by organizational structures or management systems [55,56], and a strong leadership [52,54,55,56] facilitated IPC. At the inter-individual level, facilitators included reinforcing or creating a strong team cohesion by improving communication between professionals, clarifying roles and responsibilities, and creating a trusting environment to value each professional’s skills [52,53,54,55,56].

## Discussion

The results of this overview show that, even if some specificities exist, the reported barriers were similar across the different types of collaborations, with the most frequent ones being: 1) lack of long-term funding and inadequate reimbursement policies, and payment mechanisms at the system level; 2) lack of time, insufficient training, and lack of leadership at the organizational level; 3) lack of clear role boundaries and responsibilities, poor communication, professional identity, and power issues at the inter-individual level; and 4) doubts regarding the benefits of IPC and resistance to change at the individual level. In contrast, facilitators varied depending on the type of IPC, suggesting that reported facilitators were more context-specific than barriers. The most reported facilitators related to: 1) available funding, supportive policies, incentives and compensations for professionals at the system level; 2) reorganizing practices and team structure, co-location, tools for care processes, and providing training and sufficient human resources at the organizational level; 3) the quality of communication, the respect and cohesion between professionals and a shared power at the inter-individual level; and 4) a positive attitude toward IPC at the individual level.

We observed that barriers and facilitators at organizational and inter-individual levels were particularly prominent in included reviews and across all types of IPC, in contrast to the system-level. These latter aspects were potentially less sought-after in interventional studies that aimed to improve process or health outcomes and had received funding for this purpose. It is thus likely that they focused on what worked or not at the level of their intervention rather than at the system-level. Yet system-level factors, and in particular the possibility of the health system to sustainably fund IPC, are critical for some types of collaboration such as primary care teams, collaborative care models, and PCP-nurse collaboration. Integrating nurses into the PCPs’ practices can be a major challenge in terms of funding, particularly in countries where PCPs are generally independent small-business owners, and function on a Fee-For-Service (FFS) model. In such contexts, it is in fact more convenient for PCPs to develop a collaboration outside the practice but with the risk of losing quality in collaboration [57].

The lack of multidisciplinarity in healthcare professionals’ education and training was another system-level factor under-reported in the included reviews, most likely because it may represent a distal cause not immediately perceptible to the actors. However, scholars agree that it is an indispensable prerequisite for the adoption of IPC by healthcare professionals in the future [58]. More specifically, the creation of a common culture around IPC, a main goal of interprofessional education [59] and a key aspect to maintain collaborations [60], should be encouraged and promoted at the system level. Currently, efforts are made to enhance interprofessional education during under- and graduate studies [61,62]. Even though the latter is essential to change future professionals’ perception and practice of IPC, similar efforts should also be invested in continuing professional education and training.

When system-level actions are essential to increase the recognition of the roles of healthcare professionals and legitimize IPC [46], they must be complemented by organizational and inter-individual level changes to favor professionals’ acceptance and embracement. For example, a policy analysis conducted in Ontario (Canada) [63] reported that a legislative support favoring IPC did not suppress important barriers. Moreover, since the implementation of IPC through a top-down approach increased professionals’ impression of an injunction to collaborate, several reviews underlined the importance of using bottom-up strategies to tailor IPC to its context and favor its acceptance by professionals [11,31,37,55].

At the organizational level, human resource limitations, particularly lack of time, were a major concern in all types of collaboration. Implementing organizational changes, such as team reorganization and coordination, or provision of efficient tools, requires not only energy and time but also skills that professionals may not have. Coaching strategies have thus recently emerged in primary care to guide interprofessional teams on organizational aspects [64]. A strong facilitator of IPC that was mentioned in the majority of included reviews and across all types of collaboration is co-location. This is not a surprise, as it has often been cited as a key enabler of collaborative work in the literature [65,66,67], by not only facilitating communication but also reducing power imbalances between professionals [68].

Regarding inter-individual factors, results show that perceived threats to professional identity, role definition and poor communication represent central challenges for IPC in primary care. In fact, these barriers are crucial in primary care where IPC requires strong and effective teamwork [69], even though professionals are less used to adopting a team-based functioning [70]. If role definition and poor communication seemed to be of concern in all types of IPC, fear of losing one’s professional identity was not reported in all types of IPC. This was particularly true regarding PCP-nurse and PCP-pharmacist collaboration, where PCPs have to accept to delegate or even transfer activities to other professionals. Moreover, PCPs feel that the involvement of other healthcare professionals in their patients’ management may jeopardize, or even hinder, relational continuity, a fundamental tenet of primary care [13,24,71]. In addition, IPC may be difficult to implement in a context where traditional hierarchies between disciplines persist. Actually, compliance with the medical hierarchy could result in power imbalances in collaborative teams [72], and lead to non-inclusive decision-making processes, poor communication and coordination issues [73]. Unfortunately, issues related to professional identity are difficult to address because they are often rooted in power struggles [68]. In fact, some authors have attempted to develop and conceptualize an interprofessional identity that could replace the existing identities of each professional group involved in IPC [74,75]. However, even if there is a rising interest towards developing such an identity, its conceptualization and the way it could be promoted among healthcare professionals remains unclear [76]. Other authors have suggested to focus on team functioning by promoting shared roles and leadership, or by adopting open communication. For example, role boundaries defined by separate lines of management and lacking flexibility are known to decrease teamwork effectiveness in primary care [70,77]. Thus, efforts could target flexibility and shared leadership between professionals [78]. In the long term, however, the most promising option seems to rely on education, and more particularly on mentoring [68].

The main strength of our review is that we comprehensively examined and summarized barriers and facilitators of IPC in the primary care setting, using state-of-the-art methodology. Nevertheless, results need to be interpreted according to the following limitations. First, our two main concepts (primary care and IPC) are not consensually defined in the literature. The operational definition used for the literature search and identification of reviews could have led us to miss some reviews. Also, the search strategy and eligibility criteria were not defined specifically to find barriers and facilitators, but according to the larger objective of the overview to include a wide range of articles on IPC in primary care, which means that we have also incorporated data from reviews that were not primarily intended to study barriers and facilitators of IPC. Second, it was not possible to distinguish barriers and facilitators to implementing IPC from barriers and facilitators to practicing IPC since they were most of the time not differentiated in the included reviews. These were considered jointly despite the fact that some barriers or facilitators may be more specific to one or the other phenomenon. Third, even though thematic synthesis of our data allowed the identification of clearly prominent themes in an organized and structured way, it offers little possibility to develop thematic categories of higher order beyond those identified in the literature [79]. Fourth, we were confronted to the common challenge of study overlap when conducting overviews [27]. Even though the Corrected Covered Area (CCA) suggested only a slight overlap between the 29 reviews, some primary studies were included in more than one review. Therefore, we cannot exclude that some study results, overrepresented, may have biased the overall depiction of barriers and facilitators. Overlap between the six types of IPC is also possible. In fact, the latter were not mutually exclusive, which is due to an overlap between the scopes of the reviews themselves. Finally, it appeared that more than half of the included reviews presented a high risk of bias. Despite the fact that the ROBIS tool can be used when assessing the quality assessment of different types of reviews, it was not specifically designed for qualitative and mixed methods reviews. Therefore, the quality assessment of the included reviews should be interpreted with caution.

## Conclusion

Despite some specificities according to the types of collaboration, the most often cited barriers and facilitators were reported across different contexts and intervened mostly at the organizational and inter-individual levels. It can be expected that the barriers identified at the system and individual levels will be gradually overcome with the broader implementation of interprofessional education and the setting up of collaborative projects and practices at the local level. In fact, governance, professional practices and attitudes are closely linked, and will evolve together as IPC becomes more and more familiar in primary care. We believe that this overview of reviews, by identifying the most prominent barriers and facilitators to IPC in primary care, can prove useful for the development of tools to guide decision-makers in the implementation of interprofessional collaboration.

## Additional File

The additional file for this article can be found as follows:

10.5334/ijic.5589.s1 Supplementary material. Table S1–S4.
