# Supplementary material for: An Overview of Reviews on Interprofessional Collaboration in Primary Care: Barriers and Facilitators
Source: Int J Integr Care. 2021 Jun 22;21(2):32. doi: 10.5334/ijic.5589 (PMC8231480; doi:10.5334/ijic.5589)
Supplement: Supplementary material. — Table S1–S4. [file ijic-21-2-5589-s1.pdf]

## Table S1: search strategy details

| | |
|---|---|
| **Medline Ovid** - Ovid MEDLINE(R) and Epub Ahead of Print, In-Process & Other Non-Indexed Citations and Daily | **(1)** (Cooperative Behavior/ or Interprofessional Relations/ or Physician-Nurse Relations/ or Interdisciplinary Communication/ or Patient Care Team/ or (collaborati* or cooperati* or co-operati* or crossdisciplinar* or cross-disciplinar* or integrated care or interdisciplin* or inter-disciplin* or interprofession* or inter-profession* or multidisciplin* or multi-disciplin* or multiprofession* or multi-profession* or team* or transdisciplin* or trans-disciplin*).ti,ab,kf.)<br>AND<br>**(2)** (Community Health Centers/ or Community Health Services/ or Community Health Nursing/ or Community Mental Health Services/ or Community Pharmacy Services/ or Home Care Services/ or General Practice/ or Family Practice/ or General Practitioners/ or Physicians, Family/ or Physicians, Primary Care/ or Primary Health Care/ or Office Visits/ or Primary Care Nursing/ or (((community or primary) adj3 (care or health or healthcare or practitioner*)) or (community based medicine or (communit$3 adj5 nurse?) or community nursing or community pharmac* or family doctor* or family medicine or family physician* or family medical practice* or family practi* or GP or GPs or general medical practice or general medicine or general physician* or general practi* or health center? or health centre? or medical home* or primary practice)).ti,ab,kf.<br>AND<br>**(3)** **(**meta-analysis/ or meta-analysis as topic/ or (meta analy* or metanaly* or metaanaly* or meta regression).ti,ab. or ((systematic* or evidence*) adj3 (review* or overview*)).ti,ab. or (search strategy or search criteria or systematic search or study selection or data extraction).ab. or (search* adj4 literature).ab. or (concept synthesis or conceptual review or critical interpretive synthesis or framework synthesis or integrative review or integrative literature review or literature review or meta-data-analysis or meta-ethnography or (meta adj2 narrative) or meta-study or meta-synthesis or mixed method* review or mixed research synthesis or mixed studies review or narrative review or narrative synthesis or realist review or realist synthesis or scoping review or scoping study or qualitative evidence synthesis or qualitative interpretive meta-synthesis or qualitative research synthesis or qualitative systematic review or thematic synthesis or theoretical synthesis).ti,ab.) |
| **PubMed** | **(1)** ((collaborati*[tiab] or cooperati*[tiab] or co-operati*[tiab] or crossdisciplinar*[tiab] or cross-disciplinar*[tiab] or integrated care[tiab] or interdisciplin*[tiab] or inter-disciplin*[tiab] or interprofession*[tiab] or inter-profession*[tiab] or multidisciplin*[tiab] or multi-disciplin*[tiab] or multiprofession*[tiab] or multi-profession*[tiab] or team*[tiab] or transdisciplin*[tiab] or trans-disciplin*[tiab]) |

| | |
|---|---|
| | AND<br>**(2)** (community based medicine[tiab] or community care[tiab] or community health[tiab] or community healthcare[tiab] or community mental health service*[tiab] or community mental health nursing[tiab] or community nurse[tiab] or community nurses[tiab] or community nursing[tiab] or community pharmac*[tiab] or community practitioner*[tiab] or family doctor*[tiab] or family medicine[tiab] or family physician*[tiab] or family medical practice*[tiab] or family practi*[tiab] or GP[tiab] or GPs[tiab] or general medical practice[tiab] or general medicine[tiab] or general physician*[tiab] or general practi*[tiab] or health center[tiab] or health centers[tiab] or health centre[tiab] or health centres[tiab] or medical home*[tiab] or primary care[tiab] or primary health[tiab] or primary healthcare[tiab] or primary practice[tiab] or primary practitioner*[tiab])<br>AND<br>**(3)** (meta analy*[tiab] or metanaly*[tiab] or metaanaly*[tiab] or meta regression[tiab] or evidence overview*[tiab] or evidence review*[tiab] or systematic overview*[tiab] or systematic review*[tiab] or search strategy[tiab] or search criteria[tiab] or systematic search[tiab] or study selection[tiab] or data extraction[tiab] or literature search*[tiab] or search literature[tiab] or concept synthesis[tiab] or conceptual review[tiab] or critical interpretive synthesis[tiab] or framework synthesis[tiab] or integrative review[tiab] or integrative literature review[tiab] or literature review[tiab] or meta-data-analysis[tiab] or meta-ethnography[tiab] or meta-narrative[tiab] or meta-study[tiab] or meta-synthesis[tiab] or mixed method review[tiab] or mixed methodological review[tiab] or mixed methodology review or mixed methods review[tiab] or mixed research synthesis[tiab] or mixed studies review[tiab] or narrative review[tiab] or narrative synthesis[tiab] or realist review[tiab] or realist synthesis[tiab] or scoping review[tiab] or scoping study[tiab] or qualitative evidence synthesis[tiab] or qualitative interpretive meta-synthesis[tiab] or qualitative research synthesis[tiab] or qualitative systematic review[tiab] or thematic synthesis[tiab] or theoretical synthesis[tiab]))<br>**NOT Medline[sb]** |
| **Embase (Embase Ovid on May 10th 2018, Embase.com on January 31st 2019)** | **(1)** ('Cooperation'/de OR 'Public Relations'/de OR 'Teamwork'/de OR 'Doctor Nurse Relation'/de OR 'Interdisciplinary Communication'/de OR (collaborati* or cooperati* or co-operati* or crossdisciplinar* or cross-disciplinar* or integrated care or interdisciplin* or inter-disciplin* or interprofession* or inter-profession* or multidisciplin* or multi-disciplin* or multiprofession* or multi-profession* or team* or transdisciplin* or trans-disciplin*):ti,ab,kw)<br>AND<br>**(2)** ('Health Center'/de OR 'Community Care'/de OR 'Community Health Nursing'/de OR 'Community Mental Health Center'/de OR 'Home Care'/de OR 'General Practice'/de OR 'General Practitioner'/de OR 'Primary Health Care'/de OR 'Primary Medical Care'/de |

| | |
|---|---|
| | OR (('community' or 'primary') NEAR/3 ('care' or 'health' or 'healthcare' or 'practitioner*')):ti,ab,kw OR 'community based medicine':ti,ab,kw OR (communit* NEAR/5 nurse*):ti,ab,kw OR ('community nursing' or 'community pharmac*' or 'family doctor*' or 'family medicine' or 'family physician*' or 'family medical practice*' or 'family practi*' or 'GP' or 'GPs' or 'general medical practice' or 'general medicine' or 'general physician*' or 'general practi*' or 'health center*' or 'health centre*' or 'medical home*' or 'primary practice'):ti,ab,kw)<br>AND<br>**(3)** ('Meta analysis'/de OR 'Meta analysis (topic)'/de OR 'Systematic review'/de OR 'Systematic review (topic)'/de OR ('meta analy*' or 'metanaly*' or 'metaanaly*' or 'meta regression'):ti,ab OR (('systematic*' or 'evidence*') NEAR/3 ('review*' or 'overview*')):ti,ab OR ('search strategy' or 'search criteria' or 'systematic search' or 'study selection' or 'data extraction'):ab OR ('search*' NEAR/4 'literature'):ab OR ('concept synthesis' or 'conceptual review' or 'critical interpretive synthesis' or 'framework synthesis' or 'integrative review' or 'integrative literature review' or 'literature review' or 'meta-data-analysis' or 'meta-ethnography' or (meta NEAR/2 narrative) or 'meta-study' or 'meta-synthesis' or 'mixed method* review' or 'mixed research synthesis' or 'mixed studies review' or 'narrative review' or 'narrative synthesis' or 'realist review' or 'realist synthesis' or 'scoping review' or 'scoping study' or 'qualitative evidence synthesis' or 'qualitative interpretive meta-synthesis' or 'qualitative research synthesis' or 'qualitative systematic review' or 'thematic synthesis' or 'theoretical synthesis'):ti,ab) |
| **CINAHL** | **(1)** MH ("Cooperative Behavior" OR "Interprofessional Relations" OR "Nurse-Physician Relations" OR "Multidisciplinary Care Team") OR TI (collaborati* OR cooperati* OR co-operati* OR crossdisciplinar* OR cross-disciplinar* OR integrated care OR interdisciplin* OR inter-disciplin* OR interprofession* OR inter-profession* OR multidisciplin* OR multi-disciplin* OR multiprofession* OR multi-profession* OR team* OR transdisciplin* OR trans-disciplin*) OR AB (collaborati* OR cooperati* OR co-operati* OR crossdisciplinar* OR cross-disciplinar* OR integrated care OR interdisciplin* OR inter-disciplin* OR interprofession* OR inter-profession* OR multidisciplin* OR multi-disciplin* OR multiprofession* OR multi-profession* OR team* OR transdisciplin* OR trans-disciplin*)<br>AND<br>**(2)** MH ("Community Health Centers" OR "Community Health Services" OR "Community Health Nursing" OR "Community Mental Health Services" OR "Home Health Care" OR "Family Practice" OR "Physicians, Family" OR "Primary Health Care" OR "Office Visits") OR TI ((community or primary) N3 (care or health or healthcare or practitioner*)) OR TI ((community based medicine or (communit*3 N5 nurse*2) or community nursing or community pharmac* or family doctor* or family medicine or family physician* or family medical practice* or family practi* or GP or GPs or general medical practice or general medicine or general |

| | |
|---|---|
| | physician* or general practi* or health center*2 or health centre*2 or medical home* or primary practice)) OR AB ((community or primary) N3 (care or health or healthcare or practitioner*)) OR AB ((community based medicine or (communit*3 N5 nurse*2) or community nursing or community pharmac* or family doctor* or family medicine or family physician* or family medical practice* or family practi* or GP or GPs or general medical practice or general medicine or general physician* or general practi* or health center*2 or health centre*2 or medical home* or primary practice))<br>AND<br>**(3)** MH ("Meta Analysis" OR "Systematic Review") OR PT ("Meta Analysis OR "Systematic Review") OR TI (meta analy* or metanaly* or metaanaly* or meta regression) OR TI ((systematic* or evidence*) N3 (review* or overview*)) OR TI ((search strategy or search criteria or systematic search or study selection or data extraction) or (search* N4 literature) or (concept synthesis or conceptual review or critical interpretive synthesis or framework synthesis or integrative review or integrative literature review or literature review or meta-data-analysis or meta-ethnography or (meta N2 narrative) or meta-study or meta-synthesis or mixed method* review or mixed research synthesis or mixed studies review or narrative review or narrative synthesis or realist review or realist synthesis or scoping review or scoping study or qualitative evidence synthesis or qualitative interpretive meta-synthesis or qualitative research synthesis or qualitative systematic review or thematic synthesis or theoretical synthesis)) OR AB (meta analy* or metanaly* or metaanaly* or meta regression) OR AB ((systematic* or evidence*) N3 (review* or overview*)) OR AB ((search strategy or search criteria or systematic search or study selection or data extraction) or (search* N4 literature) or (concept synthesis or conceptual review or critical interpretive synthesis or framework synthesis or integrative review or integrative literature review or literature review or meta-data-analysis or meta-ethnography or (meta N2 narrative) or meta-study or meta-synthesis or mixed method* review or mixed research synthesis or mixed studies review or narrative review or narrative synthesis or realist review or realist synthesis or scoping review or scoping study or qualitative evidence synthesis or qualitative interpretive meta-synthesis or qualitative research synthesis or qualitative systematic review or thematic synthesis or theoretical synthesis)) |
| **PsycINFO** | **(1)** (Cooperation/ or Collaboration/ or Interdisciplinary Treatment Approach/ or Work Teams/ or (collaborati* or cooperati* or co-operati* or crossdisciplinar* or cross-disciplinar* or integrated care or interdisciplin* or inter-disciplin* or interprofession* or inter-profession* or multidisciplin* or multi-disciplin* or multiprofession* or multi-profession* or team* or transdisciplin* or trans-disciplin*).ti,ab,id.)<br>AND<br>**(2)** (Community Health/ or Community Mental Health/ or Community Mental Health Services/ or Home Care/ or Family Medicine/ or General Practitioners/ or Family Physicians/ or Primary Health Care/ or (((community or primary) adj3 (care or health or |

| | |
|---|---|
| | healthcare or practitioner*)) or (community based medicine or (communit$3 adj5 nurse?) or community nursing or community pharmac* or family doctor* or family medicine or family physician* or family medical practice* or family practi* or GP or GPs or general medical practice or general medicine or general physician* or general practi* or health center? or health centre? or medical home* or primary practice)).ti,ab,id.)<br>AND<br>**(3)** (Meta Analysis/ or (meta analy* or metanaly* or metaanaly* or meta regression).ti,ab. or ((systematic* or evidence*) adj3 (review* or overview*)).ti,ab. or (search strategy or search criteria or systematic search or study selection or data extraction).ab. or (search* adj4 literature).ab. or (concept synthesis or conceptual review or critical interpretive synthesis or framework synthesis or integrative review or integrative literature review or literature review or meta-data-analysis or meta-ethnography or (meta adj2 narrative) or meta-study or meta-synthesis or mixed method* review or mixed research synthesis or mixed studies review or narrative review or narrative synthesis or realist review or realist synthesis or scoping review or scoping study or qualitative evidence synthesis or qualitative interpretive meta-synthesis or qualitative research synthesis or qualitative systematic review or thematic synthesis or theoretical synthesis).ti,ab.) |
| **Cochrane Database of Systematic Reviews + Database of Abstracts of Reviews of Effect (DARE)** | **(1)** ((collaborati* or cooperati* or co-operati* or crossdisciplinar* or cross-disciplinar* or integrated care or interdisciplin* or inter-disciplin* or interprofession* or inter-profession* or multidisciplin* or multi-disciplin* or multiprofession* or multi-profession* or team* or transdisciplin* or trans-disciplin*)<br>AND<br>**(2)** (((community or primary) near/3 (care or health or healthcare or practitioner*)) or (community based medicine or (communit* near/5 nurse?) or community nursing or community pharmac* or family doctor* or family medicine or family physician* or family medical practice* or family practi* or GP or GPs or general medical practice or general medicine or general physician* or general practi* or health center? or health centre? or medical home* or primary practice))):ab,ti |
| **JBI Database of Systematic Reviews and Implementation Reports** | **(1)** (Cooperative Behavior/ or Interprofessional Relations/ or Physician-Nurse Relations/ or Interdisciplinary Communication/ or Patient Care Team/ or (collaborati* or cooperati* or co-operati* or crossdisciplinar* or cross-disciplinar* or integrated care or interdisciplin* or inter-disciplin* or interprofession* or inter-profession* or multidisciplin* or multi-disciplin* or multiprofession* or multi-profession* or team* or transdisciplin* or trans-disciplin*).ti,hw,sa.)<br>AND<br>**(2)** (Community Health Centers/ or Community Health Services/ or Community Health Nursing/ or Community Mental Health Services/ or Community Pharmacy Services/ or Home Care Services/ or General Practice/ or Family Practice/ or General |

| | |
|---|---|
| | Practitioners/ or Physicians, Family/ or Physicians, Primary Care/ or Primary Health Care/ or Office Visits/ or Primary Care Nursing/ or community health.sa. or (((community or primary) adj3 (care or health or healthcare or practitioner*)) or (community based medicine or (communit$3 adj5 nurse?) or community nursing or community pharmac* or family doctor* or family medicine or family physician* or family medical practice* or family practi* or GP or GPs or general medical practice or general medicine or general physician* or general practi* or health center? or health centre? or medical home* or primary practice)).ti,hw,sa.)<br>AND<br>**(3)** (meta-analysis/ or meta-analysis as topic/ or (meta analy* or metanaly* or metaanaly* or meta regression).ti,tx. or ((systematic* or evidence*) adj3 (review* or overview*)).ti,tx. or (search strategy or search criteria or systematic search or study selection or data extraction).tx. or (search* adj4 literature).tx. or (concept synthesis or conceptual review or critical interpretive synthesis or framework synthesis or integrative review or integrative literature review or literature review or meta-data-analysis or meta-ethnography or (meta adj2 narrative) or meta-study or meta-synthesis or mixed method* review or mixed research synthesis or mixed studies review or narrative review or narrative synthesis or realist review or realist synthesis or scoping review or scoping study or qualitative evidence synthesis or qualitative interpretive meta-synthesis or qualitative research synthesis or qualitative systematic review or thematic synthesis or theoretical synthesis).ti,tx. or ("systematic review protocols" or systematic reviews).pt.) |
| **PROSPERO** | **(1)** ((collaborati* or cooperati* or co-operati* or crossdisciplinar* or cross-disciplinar* or integrated care or interdisciplin* or inter-disciplin* or interprofession* or inter-profession* or multidisciplin* or multi-disciplin* or multiprofession* or multi-profession* or team* or transdisciplin* or trans-disciplin*)<br>AND<br>**(2)** (community based medicine or community care or community health or community healthcare or community mental health service* or community mental health nursing or community nurse or community nurses or community nursing or community pharmac* or community practitioner* or family doctor* or family medicine or family physician* or family medical practice* or family practi* or GP or GPs or general medical practice or general medicine or general physician* or general practi* or health center or health centers or health centre or health centres or medical home* or primary care or primary health or primary healthcare or primary practice or primary practitioner*)):CM,CS,CT,IV,OP,PA,RQ,SM,TI |
| **EPISTEMONIKOS** | **(1)** title:(collaborati* or cooperati* or co-operati* or crossdisciplinar* or cross-disciplinar* or integrated care or interdisciplin* or inter-disciplin* or interprofession* or inter-profession* or multidisciplin* or multi-disciplin* or multiprofession* or multi-profession* or team* or transdisciplin* or trans-disciplin*) |

| | |
|---|---|
| | AND<br>**(2)** title:("community based medicine" OR "community care" OR "community health" OR "community healthcare" OR "community mental health service" OR "community mental health services" OR "community mental health nursing" OR "community nurse" OR "community nurses" OR "community nursing" OR "community pharmacy" OR "community pharmacies" OR "community practitioner" OR "community practitioners" OR "family doctor" OR "family doctors" OR "family medicine" OR "family physician" OR "family physicians" OR "family medical practice" OR "family medical practices" OR "family practice" OR "family practitioner" OR "family practitioners" OR GP OR GPs OR "general medical practice" OR "general medicine" OR "general physician" OR "general physicians" OR "general practice" OR "general practitioner" OR "general practitioners" OR "health center" OR "health centers" OR "health centre" OR "health centres" OR "medical home" OR "medical homes" OR "primary care" OR "primary health" OR "primary healthcare" OR "primary practice" OR "primary practitioner" OR "primary practitioners") |

## Table S2: Data extraction form

| **Review details** (indicate page where information was found) | | |
|---|---|---|
| **Study ID** (First_author Year) | | |
| **Title** | | |
| **Published protocol** | ☐ yes *(see attached)* | ☐ no |
| **Review objectives** | | |
| **Type of review** | **A review that seeks to include:**<br>☐ Qualitative studies<br>☐ Quantitative studies<br>☐ Mixed methods studies<br>☐ Conceptual / theoretical studies | Other:<br>☐ Not specified |
| | Type of review, as cited by authors (e.g. "narrative review", "integrative review", "qualitative synthesis"): | |
| **Population of review** | **Restrictions on patients characteristics:**<br>☐ age:<br>☐ condition:<br>☐ gender/ethnicity/other: | ☐ No restrictions in patient characteristics |
| **Context / setting of review** | **Definition/description of the primary care setting:**<br><br>**The primary studies of the review includes the following settings:**<br>☐ GP offices / office-based practices (☐ solo, ☐ group)<br>☐ Community health centers<br>☐ Primary health care practices<br>☐ Patient-centered medical home (PCMH)<br>☐ Across settings: interface between primary care and secondary/tertiary/community care/other organizations services<br>☐ "Primary care" (unspecified)<br>☐ "Community care" (unspecified)<br>☐ Other: | |
| | **Geographic boundary of the review:** | ☐ No geographic boundary |
| **Definition of IPC / description of the intervention** | **Definition of IPC** (theoretical and/or operational) **/ of the intervention**: | |

| | | | |
|---|---|---|---|
| | **Typology of collaboration**<br>☐ Collaboration <u>within</u> primary care practices/institutions<br>☐ Collaboration <u>between</u> primary care provider(s) and other healthcare professional(s) working outside the primary care sector<br><br>☐ Dyad (e.g. GP-nurse, GP-pharmacist)<br>☐ More than 2 health disciplines | | |
| | Notes: | | |
| **Outcomes assessed** | ☐ Barriers and/or facilitators<br>☐ Effect of IPC on quality of care (process and/or outcome)<br>    ☐ … at the patient level<br>    ☐ … at the healthcare professional level<br>    ☐ … at the organizational level (other than cost)<br>    ☐ cost-effectiveness<br>☐ Theoretical models, typologies or conceptual frameworks | | |
| | More precisely: | | |
| **Search strategy and methods** | | | |
| **Sources / database searched** | ☐ Medline<br>☐ Embase<br>☐ Cinahl<br>☐ PsycINFO<br>☐ CENTRAL<br>☐ HealthSTAR | ☐ ProQuest<br>☐ Web of Science<br>☐ Scopus<br>☐ Pascal<br>☐ Cochrane library<br>☐ JBI EBP database | ☐ Lists of references<br>☐ Grey literature<br>☐ Other:<br>☐ Other:<br>☐ Other:<br>☐ Other: |
| **Search restrictions** (language, years, region, etc.) | | | |
| **Other eligibility criteria** | | | |
| **Instrument / tool used for quality appraisal of studies** | | | |
| ***Characteristics of included studies* table** | ☐ Yes: Table n°:<br>Additional file n°: | | ☐ No |
| **Method of analysis / synthesis of results** | ☐ Narrative synthesis:<br>☐ Thematic analysis:<br>☐ Taxonomic analysis: | | Presentation of results supported by:<br>☐ Tabulation<br>☐ Framework/model |

| | | |
|---|---|---|
| | ☐ Other qual. analysis:<br>☐ Meta-analysis:<br>☐ Other quant. analysis: | ☐ Forest plot<br>☐ Other: |
| **Details of primary studies** | | |
| **Study selection** | Number of records screened:<br>Number of records excluded with reasons:<br>Number of studies included (qualitative analysis):<br>Number of studies included (quantitative analysis):<br>Flow diagram: ☐ Yes, Figure n°:<br>☐ No | |
| **Range of included studies** (years) | | |
| **Study design of included studies** | ☐ Qualitative studies<br>☐ Quantitative studies<br>☐ Mixed methods studies<br>☐ Conceptual / theoretical studies | |
| | ☐ RCT<br>☐ Q-RCT / NRCT<br>☐ CBA / BA<br>☐ ITS<br>☐ XS<br>☐ CC<br>☐ QUAL: Focus groups<br>☐ QUAL: Interviews<br>☐ QUAL: Observations<br>☐ QUANT/QUAL: Survey<br>☐ Other: | |
| **Health professionals included in IPC** (from primary studies description) | **Primary care providers**<br>☐ primary care physicians (PCP):<br>☐ family/general practitioners<br>☐ internists<br>☐ pediatricians<br>☐ geriatricians<br>☐ physician assistants<br>☐ other(s):<br>☐ primary care nurses:<br>☐ nurse practitioners<br>☐ practice nurses<br>☐ other(s): | |
| | **Other healthcare professionals**<br>☐ specialized physicians<br>☐ pharmacists<br>☐ psychologist<br>☐ mental health workers<br>☐ community psychiatric nurses<br>☐ physiotherapists<br>☐ occupational therapists<br>☐ dieticians<br>☐ midwives<br>☐ receptionists<br>☐ administrative staff<br>☐ practice managers<br>☐ public health practitioners<br>☐ public health nurses<br>☐ community nurses<br>☐ home care nurses<br>☐ social workers<br>☐ case or clinical managers<br>☐ health navigators<br>☐ health visitors<br>☐ other(s): | |

| | | |
|---|---|---|
| **Comparison groups** | ☐ Usual care<br>☐ Other intervention: | ☐ Not applicable |
| **Location of included studies** (e.g. city or region, country) | | |
| **Overall quality appraisal of included studies** | | |
| **Main results** | | |
| **Effectiveness of IPC:**<br>***patient level*** | | |
| **Effectiveness of IPC:**<br>***healthcare professional level*** | | |
| **Effectiveness of IPC:**<br>***organisation level (other than COST)*** | | |
| **COST-effectiveness** | | |
| **Factors (e.g. intervention characteristics) associated with effectiveness** | | |
| **Barriers** | | |
| **Facilitators** | | |
| **Description of models of collaboration** | | |
| **Theoretical model, typology or conceptual framework** | | |
| **Other(s) outcomes/results** | | |
| **Limitations** | | |

| | |
|---|---|
| | |
| **Other** | |
| Quality appraisal (ROBIS) | ☐Low ☐ High ☐ Unclear |
| Comments | |

## Table S3: Characteristics of included reviews on B&F of IPC implementation (n=29)[a]

| Author, year | Type of review; Synthesis method | Primary Study details: Number of included studies; Year range; Countries | Phenomena of interest/ intervention | Restriction(s) on population or setting | Quality assessment (ROBIS) | | | | |
|---|---|---|---|---|---|---|---|---|---|
| | | | | | 1. | 2. | 3. | 4. | Risk of bias in the review |
| **Interprofessional collaboration in primary care (large scope)** | | | | | | | | | |
| Belanger 2008 | Qualitative review Taxonomic analysis | n=19; 2001-2008; UK, USA, Canada, Australia, Sweden, Germany | Multi-disciplinary primary care team | Studies focusing on a specific patient population excluded | Low | Unclear | Unclear | Unclear | Unclear |
| da Conceição e Neves 2012 | Qualitative review Thematic analysis | n=10; 2000-2008; UK, Ireland, Canada, USA | Multi-disciplinary primary care team | - | Unclear | High | High | High | High |
| Morgan 2015 | Mixed methods review Thematic analysis | n=11; 2007-2013; UK, Canada, Australia, Sweden | Interprofessional collaboration (at least three individuals from at least two different disciplines) in primary care teams or involving primary care | - | Low | Low | High | Low | Low |
| Mulvale 2016 | Mixed methods review Narrative synthesis | n=9; 1999-2009; Canada, Spain, USA, Puerto Rico, UK | Interprofessional collaboration within Interprofessional Primary Care Teams (at least three different professions) | - | Low | Low | Low | High | Low |

| | | | | | | | | | |
|---|---|---|---|---|---|---|---|---|---|
| O'Reilly 2017 | Mixed methods review<br>Thematic analysis based on normalisation process theory (NPT) | n=49;<br>2004-2014;<br>Canada, USA, UK, Australia, New Zealand, Sweden, France, Spain, Netherlands, Brazil, Republic of South Africa | Interdisciplinary team in primary care | - | Low | Unclear | Unclear | Low | Unclear |
| O'Sullivan, 2015 | Mixed methods review<br>Thematic analysis | n=14;<br>2002-2012;<br>Ireland | Primary care teams | - | High | High | High | High | High |
| Sangaleti 2017 | Quantitative review<br>Pragmatic meta-aggregative approach and narrative synthesis | n=21;<br>2001-2013;<br>Brazil, Canada, USA, Ireland, New Zealand, Sweden, Lithuania, Australia. | Teamwork | - | Low | High | Low | Low | Low |
| Sorensen 2018 | Mixed methods review<br>Thematic analysis | n=19;<br>2000-2017;<br>Norway | Multi-professional collaboration between GP and other healthcare professionals in primary care | - | Low | Low | High | High | High |
| Supper, 2015 | Qualitative review<br>Thematic analysis | n=44;<br>1997-2012;<br>UK, Australia, USA, Netherlands, Canada, New Zealand, Spain, Brazil | Interprofessional collaboration (between health professionals other than nurses) | - | Low | Low | Unclear | Low | Low |

| | | | | | | | | | |
|---|---|---|---|---|---|---|---|---|---|
| Wranik, 2019 | Mixed methods review<br>Narrative synthesis based on an analytical framework | n=77;<br>2003-2017;<br>Canada, Australia, UK, New Zealand | Interprofessional primary care teams | - | Low | Low | Low | Low | Low |
| Xyrichis 2008 | Mixed methods review<br>Thematic analysis | n=10;<br>1994-2004;<br>USA,<br>UK, USA, Canada, Republic of Ireland | Interprofessional team working in primary and community care | - | High | High | High | High | High |
| **Primary care provider – mental health care provider collaboration ("Collaborative care")** | | | | | | | | | |
| Butler, 2008 | Quantitative review<br>Narrative synthesis | n=33;<br>1992-2007;<br>US | Integration of mental health services into primary care settings | Patients with mental health disorder or alcohol related substance abuse | Low | Low | Low | High | Low |
| Craven, 2006 | Quantitative review<br>Narrative synthesis | n=38;<br>1992-2005;<br>USA, UK, Australia | Collaboration between primary care providers and mental health providers | - | Low | High | High | High | High |
| Dham, 2017 | Mixed methods review<br>Narrative synthesis | n=29;<br>1994-2016;<br>USA, UK, Australia, Canada | Collaborative care interventions in primary mental health care | Older adults with psychiatric disorders | Low | High | High | High | High |
| Fuller, 2011 | Mixed method review<br>Thematic analyses | n=30;<br>1999-2009;<br>N/A | Primary mental health care linkage (connection between two or more professionals, | Primary mental health care | High | Low | High | Unclear | High |

| | | | | | | | | | |
|---|---|---|---|---|---|---|---|---|---|
| | | | including a primary care provider) | | | | | | |
| Overbeck, 2016 | Quantitative review Meta-analysis | n=17; 2003-2015; USA, UK, Canada | Collaborative care interventions in primary mental health care | Adult patients with depression or anxiety | Low | Low | Low | Low | Low |
| Wood, 2017 | Mixed methods review framework synthesis | n=18; 2003-2015; USA, UK, Canada, Germany | Collaborative care interventions in primary mental health care | Adult patients with depression | Low | Low | Low | Low | Low |
| **Physicians– nurse collaboration in primary care** | | | | | | | | | |
| McInnes, 2015 | Qualitative review Thematic analysis | n=11; 2000-2013; New Zealand, Australia, Canada, Lithuania, France, Germany | Collaboration between GP and nurses in general practice | - | Low | High | Unclear | Low | High |
| Schadewaldt, 2013 | Mixed methods review Thematic synthesis | n=27; 1998-2012; USA, Canada, UK, Netherlands, Sweden, Ireland, New Zealand | Collaboration between medical practitioners and nurse practitioners in collaborative practice | - | Low | High | High | Low | High |
| **Primary care provider – specialty care provider collaboration** | | | | | | | | | |
| Carmont, 2017 | Mixed methods review Narrative synthesis | n=17; 2001-2014; Australia, UK, Canada, Denmark, Netherlands | Integration/shared care between GP and specialist secondary clinician/service in palliative care | Palliative care | Low | High | Low | Low | Low |
| Dossett, 2017 | Mixed methods review Thematic analysis | n=36; 2001-2015; USA, UK, Australia, New Zealand, Netherlands | Relationship and communication between PCP and cancer specialists | Cancer care | Low | High | Unclear | High | High |

| | | | | | | | | | |
|---|---|---|---|---|---|---|---|---|---|
| Mitchell, 2015 | Quantitative review Narrative synthesis | n=10; 2002-2010; New Zealand, Australia, UK, USA, Belgium, Ireland | Integrated models delivered at the primary-secondary interface (with direct interaction between primary and secondary care providers) | Adults patients with chronic conditions | High | Low | High | High | High |
| **Primary care provider – pharmacist collaboration** | | | | | | | | | |
| Bardet, 2015 | Mixed methods review Thematic analysis | n=16; 2001-2013; USA, UK, Australia | Collaboration between primary care provider and community pharmacist | - | Low | Unclear | Low | Unclear | Unclear |
| Bollen, 2019 | Mixed methods review Narrative synthesis with thematic analysis | n=37; 2003-2017; Australia, Canada, UK, Germany, Ireland, Iran, Malaysia, Scotland, Spain, USA, Wales, Cuba | Interprofessional collaboration between GP and community pharmacists | - | Low | High | High | Low | High |
| **Intersectoral collaboration** | | | | | | | | | |
| Davies, 2011 | Mixed methods review Narrative synthesis, framework analysis | n=17; 1998-2008; UK, Australia, USA, Sweden | Integration between health care professionals and care home staff | Nursing homes | Low | Low | Low | Low | Low |
| Harnagea, 2017 | Mixed methods review Thematic analysis | n=58; 1987-2016; USA, Australia, Canada, France, Sweden, Norway, Switzerland, Nepal, Bangladesh, Indonesia, | Integration of oral health into primary care | - | Low | Low | High | High | High |

| | | | | | | | | | |
|---|---|---|---|---|---|---|---|---|---|
| | | Tanzania, Nigeria, Thailand, Peru, Brazil, New Zealand, UK and Iran | | | | | | | |
| Kirst, 2017 | Mixed methods review Realist approach | n=65; 1985-2015; USA, UK, Canada, Australia, the Netherlands, Italy, France, Sweden and New Zealand. | Multidisciplinary teams in integrated care programs | Older adults with complex needs (muldimorbidity) | Low | Low | Low | Low | Low |
| Leenars, 2015 | Mixed methods review Narrative synthesis | n=40; 2000-2104; Canada, USA, Netherlands, Australia, UK, Scotland, Germany, Sweden, Colombia, UK. | Collaboration between primary care and sport sector (intersectoral collaboration) | - | High | High | Low | Low | High |
| Martin-Misener, 2012 | Mixed methods review Thematic analysis | n=114; 1989-2008; UK, USA, Canada, Australia, New Zealand, Finland | Collaboration between primary care and public health (interorganizational collaboration) | - | Low | Low | High | High | High |
| [a]GP: General practitioners; PCP: Primary care providers | | | | | | | | | |

## Table S4: Detailed results of included reviews for barriers and facilitators (n=29)[a]

| Author, year | Main results (Barriers and Facilitators) | Level | | | |
|---|---|---|---|---|---|
| | | System | Organisat. | Inter-indiv. | Individual |
| **Interprofessional collaboration in primary care (large scope)** | | | | | |
| Supper, 2015 | **Barriers:**<br><br>**<u>Principal barriers</u>**:<br>• Challenges of definition<br><br>• Lack of awareness of others' roles and competences<br>• Lack of shared information, confidentiality and responsibility, team building and interprofessional training, long-term funding and joint monitoring.<br><br><u>Pharmacists</u>:<br>• Lack of mandate for pharmacists' evolving roles at a logistical (time, financial support) and team level (relationship building)<br>• Potential conflicts of interest could induce a lack of legitimacy, increased by an existing 'public–private' conflict with GP<br>• Pharmacists' medico-legal responsibility placed limits on the extension of their roles to diagnosis and prescription.<br>• Lack of clinical information and possible threats to confidentiality<br>• Lack of training or skills was an issue for some pharmacists.<br><u>Mental health providers</u>:<br>• Attitudinal barriers linked to concepts specific to the team members (such as informal VS formal communication, physician VS patient responsibility, holistic client, focused VS illness-focused model<br>• Expected roles (including care functions, teaching and supporting team)<br>• Concerns about role ownership, attachment to maintaining continuous relationships with patients and lack of clear rules for professionals to be consulted<br>• Covert barriers, including financial, geographical and time constraints | ✓ | ✓ | ✓ | ✓ |

| Author, year | Main results (Barriers and Facilitators) | Level | | | |
|---|---|---|---|---|---|
| | | System | Organisat. | Inter-indiv. | Individual |
| | <u>Midwives</u>:<br>• Lack of administrative support & long term secure funding (for diversification of roles)<br>• Statutory obligations (medical abortion)<br>• GP a gatekeeper before midwives<br>• Women's preference for a doctor in maternity care<br><u>Physiotherapists</u>:<br>• Physiotherapists' lack of experience or training<br>• Insufficient public awareness of physiotherapy for self-referral<br>• Physiotherapists' responsibility of prescribing<br>• Resource implications (time, clerical support and capital investment)<br><u>Social workers</u>:<br>• Time required for case discussion<br>• Lack of space<br>• Different decision-making processes between professionals<br>• Hierarchy between GP and social workers<br>• Lack of common office or risk of over-referral<br>• Inconsistency between GP and social workers' lists, differing priorities,<br>• Lack of mutual knowledge and respect, lack of co-location for some tasks<br><u>Receptionists</u>:<br>• Emotional workload<br>• Unequal status of role as employee<br><u>Multidisciplinary teams</u>:<br>• No face-to-face interactions between professionals<br>• Poor understanding of roles and capabilities of different professionals<br>• Power relations and tendency towards boundary maintenance<br>• Lack of communication<br>• Different expectations and agendas between professionals | | | | |

| Author, year | Main results (Barriers and Facilitators) | Level | | | |
|---|---|---|---|---|---|
| | | System | Organisat. | Inter-indiv. | Individual |
| | **Facilitators**<br>• Actors’ common interest in collaboration<br>• Perceiving opportunities to improve quality of care and develop new professional fields<br><br>Pharmacists:<br>• Definition of each professional mandate guided by a professional pharmacist, based on a bottom–up approach & clear acknowledged leadership<br>• Knowledge of each other’s role<br>• Effectiveness of collaboration procedures apparent to actors<br>• Adaptation of facilities and remunerations of health professionals involved (for good communication)<br>• Intensive multidisciplinary training at both undergraduate and postgraduate level<br>Mental health providers:<br>• Flexible horizontal model for CC, adapted to multiple stakeholders’ perspectives & specific setting<br>• Patient management coherence and active participation of actors (regular and structured meetings & coordination by a local project manager)<br>• Team members’ expectations of reaching agreements and receiving regular training regarding each other’s roles<br>Midwives:<br>• Appropriate infrastructure to support diversification of roles and evaluate new roles<br>• Diversification of roles perceived as benefits for patients by professionals<br>• Shared women-centred care approach, confidentiality & proximity (medical abortion)<br>• Professional’s perceived benefits for patients<br>• Common philosophy of normality with other health professionals (maternity care)<br>Physiotherapists:<br>• Professional’s perceived benefits for patients & self-referral | ✓ | ✓ | ✓ | ✓ |

| Author, year | Main results (Barriers and Facilitators) | Level | | | |
|---|---|---|---|---|---|
| | | System | Organisat. | Inter-indiv. | Individual |
| | • Awareness of various roles<br>Social workers:<br>• On-site and full-time<br>• Benefits perceived by professionals<br>• Team awareness of skills, training & various roles of social workers<br>• Shared computer system<br>• Social worker as 'liaison care manager' in practice<br>• Global funding<br>• Joint performance monitoring of health and social care outcomes<br>• Leadership skills at the local level<br>Receptionists:<br>• GP perceived benefits of orientation<br>• Recognition of their role in triage and management of patient emotion<br>Multidisciplinary teams:<br>• Team consultations with the patient & empowerment of patient<br>• Professionals' perceived benefits | | | | |
| Wranik, 2019 | **Barriers:**<br>• Broad overarching policies or positions of official bodies (no influence on collaboration and integration)<br>• Absence of adequate funding or remuneration | ✓ | | | |
| | **Facilitators :**<br>• Co-location of providers<br>• Leadership with the right person | ✓ | ✓ | ✓ | |

| Author, year | Main results (Barriers and Facilitators) | Level | | | |
|---|---|---|---|---|---|
| | | System | Organisat. | Inter-indiv. | Individual |
| | • Bottom-up policy development, clarity and transparency in terms of goals, roles, scopes of practice and procedures<br>• Funding support<br>• Clear and explicit definitions of roles, responsibilities and scopes of practice of the various providers within a team<br>• Usefulness of developing clear protocols and processes for communication, collaboration, patient care and other aspects of teamwork and discuss importance of team meetings | | | | |
| O'Sullivan,<br>2015 | **Barriers**<br>• Need for resources to provide longer multidisciplinary consultations and stronger collaborations with mental health services<br>• Lack of GP time to fully engage in shared care with other health professionals<br>• Problems documented with GP involvement in PCT (challenges for attending PCT meetings and levels of engagement in these)<br>• Perceived lack of leadership at national level, confusion over roles and accountability<br>• Need for more understanding about required shared objectives of the PCT, and more clarity about leadership and capacity for decision making within teams<br>• Lack of understanding of the role of other professionals, problems with interprofessional communication and lack of skills required for team working<br>• Problems with skills and knowledge required to work in teams, challenges in cross-disciplinary supervision and accountability, lack of leadership training for managers | ✓ | ✓ | ✓ | |

| Author, year | Main results (Barriers and Facilitators) | Level | | | |
|---|---|---|---|---|---|
| | | System | Organisat. | Inter-indiv. | Individual |
| | • Lack of education for interprofessional working and lack of resources to support such education<br><br>• Lack of representation and/or lack of meaningful community involvement. | | | | |
| | **Facilitators**<br><br>• Information communication technologies (e.g. emails)<br><br>• Interprofessional education to develop effective knowledge and communication for team working in PC | | ✓ | ✓ | |
| O'Reilly, 2017 | **Barriers**<br><br><u>Sense making</u> *(how PC professionals make sense of team working)*<br><br>• Team reduced to a single organizational or professional framework, or driven by one profession or agenda<br><br>• Medical practitioners training and professional experience, prioritizing doctor patient dyad over collective working<br><br>• Value of shared care and responsibility of patients with other health professionals not necessarily clear to all, particularly older doctors<br><br><u>Enrolment</u> *(how PC professionals engage or 'buy in' within the team)*<br><br>• physicians did not get involved with interdisciplinary team working as easily or quickly as other professionals by continuing to work independently or withdrawing after a period of time<br><br><u>Enactment</u> *(how PC professionals enact team working)* | ✓ | ✓ | ✓ | ✓ |

| Author, year | Main results (Barriers and Facilitators) | Level | | | |
|---|---|---|---|---|---|
| | | System | Organisat. | Inter-indiv. | Individual |
| | • Mixed funding models can be problematic by undermining the trust health professionals have in each other's roles (protecting professional territory) or motivations for decisions about patient care (best treatment versus one with commercial benefit)<br><br>• Traditional hierarchies between medicine and other professions in shaping the relational and interactional dynamics<br><br><u>Appraisal</u> *(how PC professionals appraise and reflect on team working)*<br><br>• Lack of appraisal or auditing of team working | | | | |
| | **Facilitators** – *presented under the four constructs of NPT*<br><br><u>Sense making</u> *(how PC professionals make sense of team working)*<br><br>• Good understanding across health professional groups of working together<br><br>• Interdisciplinary team views team working as a potential value for their own experience and gain for patient care (experiences & outcomes)<br><br><u>Enrolment</u> *(how PC professionals engage or 'buy in' within the team)*<br><br>• Previous positive experiences of team working, current good & trusting relationship with other professionals<br><br>• Physician involvement (strong lever for driving teamwork)<br><br>• Local champions as core member of a group in driving teamwork & change, drive to galvanize network and co-ordinate team working<br><br>• Health care reform policies and initiatives from professional bodies | ✓ | ✓ | ✓ | ✓ |

| Author, year | Main results (Barriers and Facilitators) | Level | | | |
|---|---|---|---|---|---|
| | | System | Organisat. | Inter-indiv. | Individual |
| | • Possibility of financial benefit<br>• Physicians' involvement as effective champions and most resistant professional group<br><u>Enactment</u> *(how PC professionals enact team working)*<br>• Financial resources to support team composition, training opportunities, information systems for communication between professionals about administrative and clinical issues, physical space available for teamwork<br>• Clarity within team about each other's role and responsibilities<br>• Protocols for team working & interventions in the professional network to define roles<br>• Effective and regular interdisciplinary communication about patients through verbal and face-to-face communication & use of electronic medical records and electronic patient booking systems<br>• Formal & informal interactions, meetings and interactions<br>• Co-location of teams<br>• Communication based on respectful listening and acknowledgement of all professionals' contributions and expertise<br><u>Appraisal</u> *(how PC professionals appraise and reflect on team working)*<br>• evaluations (formal & informal) and informal feedback between health professionals | | | | |
| | **Barriers** | | ✓ | ✓ | |

| Author, year | Main results (Barriers and Facilitators) | Level | | | |
|---|---|---|---|---|---|
| | | System | Organisat. | Inter-indiv. | Individual |
| Belanger, 2008 | <u>Strategies for Organizational Change toward Co-operative Practice:</u><br>• Lack of time and resources limitations<br>• Ineffective systems which lead to physicians' mental exhaustion<br>• Work overload<br>• Confusion about roles, boundaries and expectations<br>• Contested boundaries in defining activities and unclear division of labor<br>• Lack of efficient communication<br>• Hierarchical structures as a barrier for team members to achieve equal roles<br><u>Dimensions of Team Interactions and Work Relations:</u><br>• Lack of trust and respect between health professionals, trust based on competencies<br>• Insufficient training<br>• GP maintaining expectations of having to provide total care in their practice, and limiting expansion of nursing roles if they feel threatened by this<br>• Delegation of work to team members accompanied by GP holding delivery of comprehensive care as an ideal<br>• GP fears loss of contact with patients and of the "complete picture"<br>• Disparate training & socialization into distinct professional groups<br>• Vaguely defined professional identities | | | | |

| Author, year | Main results (Barriers and Facilitators) | Level | | | |
|---|---|---|---|---|---|
| | | System | Organisat. | Inter-indiv. | Individual |
| | • Uncertainty over professional contribution and value | | | | |
| | **Facilitators**<br><br><u>Strategies for Organizational Change toward Co-operative Practice</u>:<br><br>• Investing time and resources towards team building<br><br>• Developing locally adapted and flexible organizational structures<br><br>• Defining clear roles and communicating effectively<br><br>• Sharing power and involving all health professionals<br><br>• Flexible and locally customized guidelines<br><br>• Development of shared goals, clinical and administrative systems to guide co-operative practice<br><br>• Common purpose or working objectives promoting team development<br><br>• Locally tailored and implemented standardization<br><br>• Use of flexible and customizable programs to support team development and suit needs of local practices<br><br>• Identification of roles of professionals and effective communication<br><br>• Routine communication devices and more formal organizational meetings to encourage feedbacks and adjustments<br><br>• Orientation session to create sense of solidarity when dealing with problems<br><br>• Share organizational power between all team members | ✓ | ✓ | ✓ | |

| Author, year | Main results (Barriers and Facilitators) | Level | | | |
|---|---|---|---|---|---|
| | | System | Organisat. | Inter-indiv. | Individual |
| | • Balance of leadership and ownership sharing<br>• Inclusion of different team members to foster feeling of contribution<br><u>Dimensions of Team Interactions and Work Relations</u>:<br>• Developing trust and respect for co-operation<br>• Maintaining central role of GP<br>• Redefining professional identities and potential for conflict<br>• Promoting team-based organizational identity<br>• Relationships of trust with other professionals for equitable distribution of workload<br>• Mutual respect<br>• Understanding of different professional roles<br>• Central role of GP in PC delivery structure<br>• Task clarification and provision of supportive training in line with emerging roles<br>• Guidance and mentoring within member's professional disciplines<br>• Use of language to reinforce group formation and belonging by ignoring internal differences and presenting a consistent group image | | | | |
| Sangaleti, 2017 | **Barriers**<br><u>Structural and ideological level</u> | ✓ | ✓ | ✓ | ✓ |

| Author, year | Main results (Barriers and Facilitators) | Level | | | |
|---|---|---|---|---|---|
| | | System | Organisat. | Inter-indiv. | Individual |
| | • Patient lifestyle, health system practices, lack of staff training, hierarchy and chain of responsibility, and lack of network services such as physiotherapy and occupational therapy<br><br><u>Organizational level</u><br><br>• Work overload, power struggles in labor relations, gaps in care, efficiency, organization of practices, regulating flow of patients and the lack of professionals.<br><br><u>Relational level</u><br><br>• Professional thinks he/she owns the patient and does not talk about the case with others, lack of common goals and space to exchange ideas, lack of team cohesion<br><br><u>Others</u><br><br>• Conflicts: acquisition of knowledge and skills from other professionals, difficulties and bad communication strategies, lack of common vision, problems with physicians, referral as a source of tension, ambiguous relations between professionals, cohesion of the team, trouble understanding role of others, patient is a source of tension, conflict between professionals, struggle for power and space between professionals, tensions within the team, contribution of knowledge undervalued and not put into practice, questioning competence of nurse<br><br>• Working determinants: social division of labor, workload, different in employment contracts and wages<br><br>• Biomedical paradigm: views of professionals and patients (physicians feel responsible, division of labor in nursing teams, patients not trusting professionals other than physicians & seeking medical consultations in PC)<br><br>• Referral: difficulty in getting necessary references | | | | |

| Author, year | Main results (Barriers and Facilitators) | Level | | | |
|---|---|---|---|---|---|
| | | System | Organisat. | Inter-indiv. | Individual |
| | **Facilitators**<br><br>• Attitudes and beliefs: understanding and appreciation of teamwork, communication, confidence, humility, sense of belonging, shared responsibility, and time to listen and talk<br><br>• Roles and responsibilities: recognition of members' roles, willingness to understand and respect work of members, rethink traditional practices to handle complex cases<br><br>• Practice: integration, synergy, availability, reliability, balance between autonomy and interdependence of professions, collaboration, and responding to the patient's integral care needs.<br><br>• Communication: being open, understanding importance to listen and talk, informal meetings and frequent meetings, common language of team, explaining to other members, discussions of cases and treatments, recognition of limits to sharing information, exchanging specific information on work, consensus building, coordination of actions<br><br>• Space: access to others, face-to-face contact & discussion, sharing and sociability<br><br>• Leadership: to promote interprofessional practice, make team work together, must be shared, dependent on set of skills and influenced by traditional status of physician as leader<br><br>• Philosophies of care (comfort and connection with patients, team common goal of meeting health needs, involving family and community in the care process)<br><br>• Referral: referral to network, facilitated by effective, patient-centered relations<br><br>• Education and training: integrating teamwork as part of undergraduate study curricula | ✓ | ✓ | ✓ | ✓ |

| Author, year | Main results (Barriers and Facilitators) | Level | | | |
|---|---|---|---|---|---|
| | | System | Organisat. | Inter-indiv. | Individual |
| | • Working determinants: social division of labor, workload<br><br>• Biomedical paradigm: professionals' and patients' views about teamwork | | | | |
| Xyrichis, 2008 | **Barriers**<br><br><u>Team structure</u>:<br><br>• Team premises: team members with separate bases or buildings can result in less integration with the team, limiting team functioning and effectiveness<br><br>•Team size & composition: larger teams had lower levels of participation compared with smaller sized teams, but larger teams were externally rated by Health Authority management, the NHS parent Trust and GP, to be more effective in dimensions of clinical practice and teamworking; Status of team members may inhibit members from participating in the decision-making process and from providing input during team meetings<br><br>• Leadership: lack of clarity about leadership caused frustration, led to poor decision-making, predicted lower levels of team effectiveness & was associated with poor quality teamworking<br><br>• Moving of members to other areas<br><br><u>Organization support:</u><br><br>• Lack of organizational rewards for the team's improved working caused team members to feel concerned and disappointed with lower effectiveness over time<br><br>• Lack of organizational support to implement changes led to team members feeling powerless, discouraged<br><br><u>Team processes:</u> | | ✓ | ✓ | |

| Author, year | Main results (Barriers and Facilitators) | Level | | | |
|---|---|---|---|---|---|
| | | System | Organisat. | Inter-indiv. | Individual |
| | • Time pressure was a barrier to regular team meetings<br>• Lack of communication as causing misconceptions about each profession's roles and responsibilities<br>• Conflict related to professional identity as a barrier to positive relations in the team and effective teamwork<br><u>Clear goals and objectives</u><br>• Lack of clear understanding of each professional's role and responsibility | | | | |
| | **Facilitators**<br><u>Team structure:</u><br>• Team premises enhance information transaction, facilitate communication, and increase personal familiarity<br>• Team size & composition: larger teams appear to have lower levels of participation compared with smaller sized teams but larger teams were externally rated by Health Authority management, the NHS parent Trust and GP, to be more effective in dimensions of clinical practice and teamworking; teams with greater occupational diversity introduced more radical innovations ; teams with a high proportion of full-time staff and having worked together for longer (stability)<br><u>Organization support</u>:<br>• Organizational support both for team working and for the team's members<br>• Teams' openness to innovation and change<br>• High support for innovation | | ✓ | ✓ | |

| Author, year | Main results (Barriers and Facilitators) | Level | | | |
|---|---|---|---|---|---|
| | | System | Organisat. | Inter-indiv. | Individual |
| | <u>Team processes:</u><br><br>• Regular team meetings: associated with greater levels of innovation and increased participation of members in meeting, members considered them of high value (break down professional barriers and improve interprofessional communication), resolved interprofessional conflict and promoted positive interpersonal relations<br><br>• Climate of mutual respect and trust<br><br>• Clear goals and objectives for good team functioning by providing team with vision and enhance creativity<br><br>• Audit: evaluations sustain good performance or improve performance, regular appraisals offer a chance to discuss problems, consider appropriate solutions to improve team functioning, praise individuals for their contribution, and provide support where needed<br><br>• Effective feedback to sustain and improve their performance and provide energy and incentives to team members by giving publicity to team successes | | | | |
| da Conceição e Neves, 2012 | **Barriers**<br><br>• No common sense of what participants understood as 'teams'<br><br>• Lack of a set of common values to work on<br><br>• Lack of knowledge of skills needed for shared care<br><br>• Hierarchical practices, lack of common goals and inability to communicate<br><br>• Power differences in the team<br><br>• Insecurity about implications for professional identity | | ✓ | ✓ | ✓ |

| Author, year | Main results (Barriers and Facilitators) | Level | | | |
|---|---|---|---|---|---|
| | | System | Organisat. | Inter-indiv. | Individual |
| | • Other professionals in a health team do not always have knowledge about functions and scope of nurses' practice, or knowledge is superficial (contributes to perception of role based on decontextualized stereotypes that influence team dynamics)<br><br>• Poor appropriation of concept of care | | | | |
| | **Facilitators**<br><br>• Organizational support for change, leadership, commitment and broad participation<br><br>• Training program to support teamwork and role development<br><br>• Team leader for collective performance & personal growth and development, to remove obstacles to effective teamwork, facilitate communication and promote decision making<br><br>• Development management, assertiveness and trust competencies<br><br>• Interprofessional learning (often informal through interactions in context of practice)<br><br>• Understanding role of others<br><br>• Respect for functions/role<br><br>• Common understanding of purpose, philosophy and principles of PC, enabling a clarification of responsibilities, competencies and functions<br><br>• Communication among different professionals<br><br>• Joint training and identification of facilitators | | ✓ | ✓ | ✓ |

| Author, year | Main results (Barriers and Facilitators) | Level | | | |
|---|---|---|---|---|---|
| | | System | Organisat. | Inter-indiv. | Individual |
| | • Team reinforcement for development of new forms and roles clarity of objectives, leadership, commitment and broad participation of all professional groups | | | | |
| Morgan, 2015 | **Facilitators/barriers**<br><br>• Importance of a multi-level approach to achieve frequent shared informal communication<br><br>Top down organizational factors acting as facilitators/ barriers to IPC<br><br>• Practice policy and structure, organizationally endorsed formal processes, collaborative management and leadership and opportunities for informal communication<br><br>• Need for policy and structure with PC team, requirements for formal processes (e.g. regular meetings, written documents) to allow time for teamwork and help clarify roles and responsibilities<br><br>• Management structures: explicitly collaborative, support team development and process, provide regular feedback on team performance<br><br>• Effective & supportive collaborative leadership – formalized partnerships between staff and with patients<br><br>• Creating multiple opportunities for frequent, informal communication: shared physical work space & its configuration, allowing access to essential info (mostly about patients) and ability to exchange easily and informally info & ideas, tools such as shared whiteboard in common space, common clinical records (often electronic)<br><br>Bottom-up intrinsic factors acting as facilitators/ barriers to IPC | ✓ | ✓ | ✓ | |

| Author, year | Main results (Barriers and Facilitators) | Level | | | |
|---|---|---|---|---|---|
| | | System | Organisat. | Inter-indiv. | Individual |
| | • Informal communication (brief, informal but frequent)<br><br>• Shared knowledge creation (including knowledge about teamworking) as a relational & reciprocal process & reliant on informal communication<br><br>• Shared clinical decision making achieved when common knowledge had been built | | | | |
| Sorensen, 2018 | **Facilitators**<br><br>Organizational facilitators<br><br>• Establish procedures for inter-professional meetings, documentation and handling of patient data (e.g. e-communication)<br><br>• Facilitate knowledge sharing between disconnected professionals<br><br>• Establish local, specialized multi-professional teams<br><br>• Establish system-level foundation that supports local management and leadership of MPC<br><br>Processual facilitators<br><br>• Enhance collaborative skills before introducing new professional teams, roles and responsibilities<br><br>• Develop common quality-management systems across institutions<br><br>• Allocate sufficient time for professionals to share reflections and engage in mutual learning<br><br>Relational and contextual facilitators<br><br>• Invest in professional relations that build trust, respect and continuity | ✓ | ✓ | ✓ | |

| Author, year | Main results (Barriers and Facilitators) | Level | | | |
|---|---|---|---|---|---|
| | | System | Organisat. | Inter-indiv. | Individual |
| | • Improve professionals' knowledge of each other's skills and roles through inter-professional education<br><br>• Educate patients about benefits of MPC | | | | |
| Mulvale, 2016 | **Facilitators**<br><br><u>18 factors identified as being associated with collaboration in IPCT by using the gear model:</u><br><br>• Macro factors: governance (positively associated with team climate)<br><br>• Meso factors: having formal information Systems; having an organizational Culture with respect to group culture, hierarchy, focus on efficiency and achievement<br><br>• Micro factors: team structure (having a team champion/facilitator, optimal team size); social processes (low levels of conflict in team, open communication, supportive colleagues), formal processes (setting out team vision/goals, focus on quality audit/other processes, formal recognition from supervisors, having processes for group problem-solving, having interprofessional team meetings, having decision-making processes; team attitudes (feeling part of team, support for innovation in team), individual factor (belief in interprofessional care, flexibility) | | ✓ | ✓ | ✓ |
| **Primary care provider – mental health care provider collaboration ("Collaborative care")** | | | | | |
| Butler, 2008 | **Barriers**<br><br><u>Financial</u><br><br>• Carved out mental health services | ✓ | ✓ | | ✓ |

| Author, year | Main results (Barriers and Facilitators) | Level | | | |
|---|---|---|---|---|---|
| | | System | Organisat. | Inter-indiv. | Individual |
| | • Consultation between providers not compensated<br>• Care manager not always eligible for compensation<br>• No reimbursement for two encounters on the same day with different professionals (public funding)<br>• Mental health services carved out of general medical services<br>• No reimbursement for telephone consultation<br><u>Organizational</u><br>• Resistance to change<br>• Staffing: availability of mental health specialists; acceptance of new roles<br>• Time: balancing competing demands and burden of case identification<br>• Expertise and comfort dealing with mental health problems<br>• Privacy concerns: HIPAA | | | | |
| Overbeck, 2016 | **Barriers**<br><u>Coherence</u>:<br>• Insufficient understanding of CC model, unfamiliarity with model, lack of educational programs<br><u>Cognitive participation:</u> | ✓ | ✓ | ✓ | ✓ |

| Author, year | Main results (Barriers and Facilitators) | Level | | | |
|---|---|---|---|---|---|
| | | System | Organisat. | Inter-indiv. | Individual |
| | • Lack of PCP engagement due to time pressure and competing interests in PC, problems with reimbursement, PCP being uncomfortable with diagnosing and treating mental health illness, concerns about sharing patient's private data<br><br><u>Collective action:</u><br><br>• Absence of co-location and of regular interaction<br><br>• Lack of space for additional staff<br><br>• IT systems (information technology) mostly described as hindering effective communication between actors<br><br>• Difficulty in managing mental health problems due to multifaceted nature of patients' problems (in terms of severity and/or complexity)<br><br>• Extra time needed for CC described as a problem by PCP<br><br>• Availability of funding for CC implementation<br><br><u>Reflexive monitoring:</u><br><br>• CC difficult to set up in clinics that did not have systems for monitoring patients' progress<br><br>• Absence of immediate access to objective data on patient progress | | | | |
| | **Facilitators** *(according to the 4 dimensions of NTP)*<br><br><u>Coherence:</u><br><br>• Educational programs to provide participants with principles & tools of CC model<br><br>• Use of physician champions to help professionals understand model | ✓ | ✓ | ✓ | ✓ |

| Author, year | Main results (Barriers and Facilitators) | Level | | | |
|---|---|---|---|---|---|
| | | System | Organisat. | Inter-indiv. | Individual |
| | • Clarifying roles and responsibilities between participants from primary and secondary care<br><br><u>Cognitive participation:</u><br><br>• Engagement of professionals involved<br><br>• Professional engagement strengthened by observation or communication of patients' positive outcomes in intervention<br><br>• Professional opinion leaders or local champions<br><br>• Covering PCP operating costs related to CC helped improve engagement<br><br><u>Collective action:</u><br><br>• Co-location of care manager (CM) and PCP in PC clinic by increasing regular face-to-face interactions (formal & informal)<br><br>• Interactions centered on patient cases<br><br>• Face-to-face referral between professionals<br><br>• Professional & social skills of CM<br><br>• Educational programs which prepare CM for their organizational position<br><br>• Professionals engaging with patients (empathy, language, starting out with simple treatment strategies)<br><br>• Standardized instruments for including patients in CC, keeping track of progress and planning support | | | | |

| Author, year | Main results (Barriers and Facilitators) | Level | | | |
|---|---|---|---|---|---|
| | | System | Organisat. | Inter-indiv. | Individual |
| | • Time and availability: model not being burdensome or creating problems with workload<br>• Implementation of CC facilitated when PCP was already used to working with other professionals such as CM<br><u>Reflexive monitoring:</u><br>• Professionals believing patients benefitted from CC<br>• Systematic feedback on patients' conditions valued by PCP; systematic monitoring enabled active follow-up and strengthened implementation of CC | | | | |
| Wood, 2017 | **Barriers to the implementation of CC:**<br><u>Multi-professional team working</u><br>• Organizations' readiness for change (physical changes to daily practice and attitudinal changes within organization's culture)<br>• Lack of strong leadership and organizational buy in<br>• Individual practitioners resistant to attitude change<br><u>Patient management plans</u><br>• Staff not understanding CC<br>• Style of material unfamiliar to staff, difficulty of introducing self-help material to patients or patients having difficulty engaging with the material<br><u>Enhanced communication</u> | ✓ | ✓ | ✓ | ✓ |

| Author, year | Main results (Barriers and Facilitators) | Level | | | |
|---|---|---|---|---|---|
| | | System | Organisat. | Inter-indiv. | Individual |
| | • Breakdowns in networks and communication pathways (poor communication between professional groups or limited technology to support timely communication)<br><br><u>Sustainability</u><br><br>• Insufficient funding to maintain program after research was conducted | | | | |
| | **Facilitators to the implementation of CC:**<br><br><u>Multi-professional team working</u><br><br>• Staff attitudes to change<br><br>• Senior physicians take role of championing service to his/her colleagues<br><br>• Strong buy in by organization with clear leadership structure led by experts in both physical and mental health<br><br><u>Case management</u><br><br>• Role of CM clearly developed and boundaries defined to all involved<br><br>• Recruiting 'right' CM with key characteristics (such as ability to learn quickly; effective communication skills; capable; being adaptable and well organized)<br><br><u>Patient management plans</u><br><br>• Structured management plans for patients, made of high-quality materials, alongside in-depth staff training delivered by confident staff<br><br>• Staff involved with sufficient training on the intervention<br><br>• Right screening and outcome tools and training all staff on these | ✓ | ✓ | ✓ | ✓ |

| Author, year | Main results (Barriers and Facilitators) | Level | | | |
|---|---|---|---|---|---|
| | | System | Organisat. | Inter-indiv. | Individual |
| | • Standardized care pathway to enhance screening (GP happier to talk about depression if they knew what to do once it was identified)<br><br>• See benefits of implemented intervention, especially behavioral activation and interventions around medication education and monitoring<br><br><u>Enhanced communication</u><br><br>• Co-location of professionals<br><br>• Use of integrated information systems (to share notes and messages)<br><br>• Supportive, constructive and regular supervision schedule (help CM deliver care and talk over difficult cases or ask questions about referral on to mental health services)<br><br><u>Scheduled follow up</u><br><br>• Scheduled follow-ups and a person ensuring they took place<br><br><u>Sustainability</u><br><br>• Funders involved from the start of the implementation to enable financial buy-in | | | | |
| Dham, 2017 | **Barriers** *(to implementation of CC)*<br><br>• Organizational resistance<br><br>• Absence of staff skills and attitude, increased workload, and lack of organizational support or funding | ✓ | ✓ | | ✓ |
| | **Facilitators**<br><br>• Screening and enhanced referral to psychiatry specialist clinics or community mental health teams | | ✓ | | |

| Author, year | Main results (Barriers and Facilitators) | Level | | | |
|---|---|---|---|---|---|
| | | System | Organisat. | Inter-indiv. | Individual |
| | • Better engagement when specialist care is collocated within PC compared with referral to specialist services<br><br>• Institutional support, presence of trained staff to facilitate collaboration process and encourage sustainability | | | | |
| Fuller, 2011 | **Barriers**<br><br><u>Clinical level</u><br><br>• Partnership formation activities: lack of common goal, differing role expectations, role expansion, referral suspicion and differences, inadequate referral information, no follow-up, link staff support, lack supervision/mentoring<br><br>• Clinician attributes: difficulty recruiting experienced mental health workers to collaborative roles<br><br>• Clinic physical features: accommodation - inappropriate<br><br>• Organizational level: lack of resources & no change management, leadership not supportive, GP anxiety about loss of control if not the leader, unclear organizational structure | ✓ | ✓ | ✓ | |
| | **Facilitators**<br><br><u>Clinical Level</u><br><br>• Partnership formation activities: joint development (joint ownership & mutually beneficial outcomes); in person active communication through regular clinical meetings, information sharing and clinical problem solving; formulation of a common understanding of the nature of partnerships, its operation and a set of agreed goals; development of processes (e.g. care planning, guideline development, referral and | | ✓ | ✓ | ✓ |

| Author, year | Main results (Barriers and Facilitators) | Level | | | |
|---|---|---|---|---|---|
| | | System | Organisat. | Inter-indiv. | Individual |
| | follow-up protocols); patient involvement in care (such as patient set self-management goals); role clarity & attention to roles of partners<br>• Link staff support: supervision, mutual support<br>• Clinician attributes: consider collaboration worthwhile & willing to take part (motivated); knowledge & skill in PC and mental health; PC focused & flexible work style, ability to fit in<br>• Clinic physical features: accommodation - visibility for interaction; larger practice size<br>• Evaluation feedback to clinicians: measurement, database & communication system (reliable patient tracking information & communication process to team)<br>• Organizational level: institutional support, leadership | | | | |
| Craven, 2006 | **Facilitators** *(for CC between PCP and mental health providers)*<br>• Preparation time, supportive structures and service reorganization<br>• Co-location of professionals<br>• Service restructuring to support changes<br>• Sufficient funding to support CC processes and practices | ✓ | ✓ | | |
| **Physician – nurse collaboration in primary care** | | | | | |
| McInnes, 2015 | **Barriers**<br>Roles and responsibilities<br>• Lack of clarity around nursing roles and scope of practice | ✓ | | ✓ | |

| Author, year | Main results (Barriers and Facilitators) | Level | | | |
|---|---|---|---|---|---|
| | | System | Organisat. | Inter-indiv. | Individual |
| | • GP territorialism by protecting own professional boundaries and expertise, particularly when roles perceived as overlapping<br>• Nurses refusing to expand their role, due to lack of clarity around their roles and responsibilities<br><u>Respect, trust and communication</u><br>• GP distrust in nurse's knowledge and skills<br>• Poor communication and exclusion from activities such as practice meetings<br><u>Hierarchy, education and liability</u><br>• Hierarchical structures, privately owned and operated small business general practices limited collaboration with GP: no nurse led board position in any of the practices, 37% of nurses attended practice meetings to address management decisions<br>• Traditional doctor's status was impetus for assuming role as team leader<br>• Difficulty of calculating true cost benefit of nurses to the small business environment of general practice<br>• GP and nurses have uni-disciplinary training (biomedical education of doctors VS experiential learning of nurses)<br>• Doctors strongly believed that nurses' education did not support their role as autonomous clinicians<br>• Potential legal implications of nurses' practice | | | | |

| Author, year | Main results (Barriers and Facilitators) | Level | | | |
|---|---|---|---|---|---|
| | | System | Organisat. | Inter-indiv. | Individual |
| | • Business model found in general practice frequently dictated power and leadership to GP | | | | |
| | **Facilitators**<br><br><u>Roles and responsibilities</u><br><br>• Clearly defined roles and shared leadership, which were skill set dependent<br><br>• GP delegating of tasks to nurses<br><br>• GP supporting nurses expanding their role in practice setting<br><br><u>Respect, trust and communication</u><br><br>• Respect, trust, professional confidence and respect for professional competence to be earned and developed<br><br>• Shared commitment to PC, open channels of communication, awareness of each profession's roles and responsibilities<br><br><u>Hierarchy, education and liability</u><br><br>• Funding structures, including those which supported patient/team encounters and salaried positions<br><br>• Nurses reported educational programs would lead to improved competencies and greater allocation of care by GP<br><br>• Nurses improved awareness of health services to the broader community and helped reduce sense of isolation experienced by solo medical practitioners<br><br>• Implementation of brief practice meetings | ✓ | ✓ | ✓ | ✓ |

| Author, year | Main results (Barriers and Facilitators) | Level | | | |
|---|---|---|---|---|---|
| | | System | Organisat. | Inter-indiv. | Individual |
| Schadewaldt, 2013 | **Barriers**<br>• Lack of awareness by MP of: NP scope of practice, level of education and what is inherent to their role<br>• Ideological differences in practice style<br>• Control issues between NP and MP<br>• MP concern about legal responsibility/liability for the care provided by NP<br>• Lack of financial support for NP role<br>• Specialists' unwillingness to accept referrals, MP lack of understanding of NP role and MP personality<br>• Imbalance of referrals between NP and MP & lack of shared care<br>• MP concern of becoming deskilled and feeling threatened by NP<br>• Limitations of NP autonomy (increased MP workload)<br>• Concerns of some MP about quality and fragmentation of care<br>• MP insecurity about advantage and disadvantage of collaborating with NP | ✓ | ✓ | ✓ | ✓ |
| | **Facilitators**<br>• NP taking over some parts of MP workload (such as education and follow-up care, 'routine cases' or patients with minor illnesses and chronic diseases); MP able to focus on more complex cases<br>• Confidence in collaborating partner's competence | ✓ | ✓ | ✓ | ✓ |

| Author, year | Main results (Barriers and Facilitators) | Level | | | |
|---|---|---|---|---|---|
| | | System | Organisat. | Inter-indiv. | Individual |
| | • Having complementary skills and similar goals with partner<br>• Previous experience of working with NP or MP and having a good relationship<br>• Reciprocity of referrals and consultations<br>• Absence of hierarchical structures and control<br>• Effective communication (face-to-face and use of technologies)<br>• Mutual trust and respect<br>• MP support in establishment of collaborative practice with NP<br>• Shared responsibility of complex cases<br>• High level of NP autonomy<br>• Working in close physical proximity or on the same site; regular meetings & collaboration<br>• Positive attitude towards collaboration<br>• Official recognition of NP role (including legal protection of professional title NP)<br>• Trust and respect shown by MP in making shared decisions<br>• MP personality<br>• Reduced waiting times for patients<br>• Complementary scope of practice between MP & NP | | | | |
| **Primary care provider – specialty care provider collaboration** | | | | | |

| Author, year | Main results (Barriers and Facilitators) | Level | | | |
|---|---|---|---|---|---|
| | | System | Organisat. | Inter-indiv. | Individual |
| Carmont, 2017 | **Barriers to engagement of GP with specialist secondary services in integration of palliative care:**<br><br>• Health system barriers: financial constraints for GP & hospital, workload, lack of standardized documents and systems, bureaucratic procedures, professional silos, lack of services and lack of infrastructure<br><br>• Process barriers: not clearly defined roles within integrated care, barriers to sharing information, health professionals' perceived lack of time, ambiguity around who should be involved, communication and technology issues<br><br>• Poor communication creating personal issues (trust, poor relationships) between relatives and patients, lack of patient or family understanding of discussions with healthcare professionals, quality of the relationship between patient and professionals, competitive or combative relationship between health professionals<br><br>• Engagement: lack of clarity of purpose (e.g. future planning, acute medical issue), participants being<br><br>unprepared (either with not receiving information, or not reading the information prior to the meeting)<br><br>• Late referral, lack of understanding of patient prognosis by family, medical professionals underestimating seriousness of patient's condition and overestimating prognosis | ✓ | ✓ | ✓ | |
| | **Facilitators to engagement of GP with specialist secondary services in integration of palliative care:**<br><br>• Effective and timely communication with appropriate level of detail and with most relevant people, receiving patient info electronically | | ✓ | ✓ | |

| Author, year | Main results (Barriers and Facilitators) | Level | | | |
| --- | --- | --- | --- | --- | --- |
| | | System | Organisat. | Inter-indiv. | Individual |
| | • Clear role definition and understanding of professionals' roles by all professionals involved<br>• Availability of GP to patient & care taker (through prompt appointments and telephone contact for medical care and referral)<br>• Family expectation that GP will be involved in medical care, family & emotional support<br>• Organized and initiated integrated care by specialist secondary service<br>• Equitable status, equal respect and authority between all participants to build partnerships<br>• Systematic method of screening patients for palliative needs<br>• Structure or framework of patient pathways with built in flexibility and real-world adaptation (including referral pathways, provision of care after hours, primary contact details and management plans)<br>• Professional development (training improving knowledge and confidence for care providers) | | | | |
| Dossett, 2017 | **Barriers**<br>Poor and delayed communication between PCP and cancer specialists<br>• Infrequent communication (PCP not sufficiently informed about patients' diagnosis or outcomes in post-referral period, PCP and oncologists reported that frequency of communication could be improved)<br>• Deficiencies in content, mode, volume and style of information transmitted by oncologists to PCP | ✓ | ✓ | ✓ | ✓ |

| Author, year | Main results (Barriers and Facilitators) | Level | | | |
|---|---|---|---|---|---|
| | | System | Organisat. | Inter-indiv. | Individual |
| | • Survivorship care plans not transmitted often enough to PCP by oncologists, limited training, no available template, and lack of reimbursement<br><br><u>Cancer specialists' endorsement of a specialist-based model of care</u><br><br>• Oncologists endorsed preference for specialist-based care because they believed patients were not well enough to go to multiple physicians<br><br>• Oncologists rarely admitted discharging cancer survivors to PCP for surveillance and survivorship<br><br>• Oncology providers indicated feeling this long-term commitment provided patients with reassurance that any potential problems would be detected as early as possible<br><br>• Oncology specialists stated struggling with discharging survivors because of the bonds established with patients, their concern for survivors' needs, and having become "emotionally invested" in patient's success (wanted to remain involved after completion of active treatment)<br><br><u>PCP role in cancer continuum</u><br><br>• PCP felt that oncologists "swallow[ed] up" the patient by providing primary and cancer care<br><br>• patient's desire for specialist care (barrier to PCP involvement)<br><br><u>Specialists and PCP uncertainty of PCP's knowledge or training to provide care</u><br><br>• Cancer specialists expressed skepticism of PCP training to provide this care<br><br>• Uncertainty regarding PCP competence to address such problems<br><br>• Lack of PCP experience or training in oncology as barriers to discharge for follow-up | | | | |

| Author, year | Main results (Barriers and Facilitators) | Level | | | |
|---|---|---|---|---|---|
| | | System | Organisat. | Inter-indiv. | Individual |
| | • Insufficient knowledge of cancer issues hinders PCP provision of survivorship care (58% of PCP in one study and 79% in another)<br>• PCP uncomfortable with patients' questions about cancer diagnosis; lack of knowledge regarding current treatment protocols<br><u>Discordant Expectations and Preferences Between Providers</u><br>• Discordance or uncertainty in provider roles, expectations and preferences in care models throughout cancer care continuum<br>• PCP and oncologists showed high discordance in perceptions of their own roles for cancer follow-up, cancer screening, and general preventive health<br>• Differing preferences among cancer specialists (medical oncologists assuming PC role in some cases, while radiation and surgical oncologists expressed a preference for only managing cancer-related issues)<br>• Preferred model of care among PCP and oncologists: 38% of PCP preferred a shared-care model compared with only 16% of oncologists<br><u>Other barriers:</u><br>• PCP thought cancer specialists excluded them because PCP might be less willing to support aggressive cancer treatments in patients with poor prognoses | | | | |
| | **Facilitators**<br><u>Communication</u><br>• EMR-generated SCP to coordinate care<br>• Centralized cancer care system with synoptic discharge communication | | ✓ | ✓ | |

| Author, year | Main results (Barriers and Facilitators) | Level | | | |
|---|---|---|---|---|---|
| | | System | Organisat. | Inter-indiv. | Individual |
| | • PCP and oncologist with a re-existing relationship and practicing in the same health care system<br><br>• Communication enhanced by integrated EMR<br><br>• Ease of reaching cancer specialists by telephone or e-mail<br><br>• Faxed, tailored chemotherapy information (compared with those who received usual correspondence)<br><br><u>PCP role in cancer continuum</u><br><br>• PCP ready to assume exclusive responsibility for survivorship care 2 to 3 years after active treatment and identified several modalities to ease transition, including a patient-specific letter from specialist, printed guidelines, expedited access to investigations when recurrence was suspected, and expedited re-referral if necessary<br><br><u>PCP Knowledge on cancer care</u><br><br>• Increased education and training, development of shared protocols, and recruitment of PC-based oncology nurses to facilitate early discharge to PCP | | | | |
| Mitchell, 2015 | **Barriers**<br><br><u>Interdisciplinary teamwork</u><br><br>• GP too busy for direct involvement in comprehensive patient care<br><br>• Inadequate access to other key personnel and lack of role clarity<br><br>• Clinicians' concerns for duplications and omissions in co-morbidities management due to lack of role clarity for referring GP and specialist clinics | | ✓ | ✓ | ✓ |

| Author, year | Main results (Barriers and Facilitators) | Level | | | |
|---|---|---|---|---|---|
| | | System | Organisat. | Inter-indiv. | Individual |
| | **Facilitators** *(of models of integrated primary-secondary care)*<br><br>Interdisciplinary teamwork<br><br>• Good coordination by personnel with an understanding of community and specialist-based care<br><br>• Clearly defined roles<br><br>Communication & information exchange<br><br>• Willingness to share information<br><br>• Supportive managerial and administrative staff<br><br>• High level of GP trust in specialists<br><br>• Improved communication between GP and hospital specialists<br><br>• Shared follow-up supported by electronic reminder systems<br><br>• Shared governance that enhanced system capacity for effective communication and collaboration<br><br>• Regular interdisciplinary team meetings that enabled information exchange<br><br>• Successful communication channels including case conferences<br><br>• Co-located GP and specialist clinics (facilitate effective communication)<br><br>• Information exchange between GP and specialists<br><br>• Ongoing access to specialists and shared follow-up<br><br>Use of shared guidelines or pathways | ✓ | ✓ | ✓ | ✓ |

| Author, year | Main results (Barriers and Facilitators) | Level | | | |
|---|---|---|---|---|---|
| | | System | Organisat. | Inter-indiv. | Individual |
| | • Pragmatic, locally agreed care protocols<br>• Protocols included: guidance for post-discharge care and review, shared care planning, patient goal-setting and self-management, structured electronic record and recall systems<br><u>Training and education</u><br>• Initial and continuing education, including postgraduate training<br>• Patient education | | | | |
| **Primary care provider – pharmacist collaboration** | | | | | |
| Bardet, 2015 | **Barriers**<br>• Lack of time and remuneration (for implementation of CPS)<br>• CP more perceived as retailers or shopkeepers by physicians, impacting evaluation of CP skills<br>• CP expressed lack of confidence in clinical knowledge and capabilities, and not self-confident enough to contact physicians | | ✓ | ✓ | |
| | **Facilitators** *(according to collaboration models)*<br><u>Key elements</u>:<br>• Trust, interdependence, perceptions and expectations about the other HCP, skills, interest for collaborative practice, role definition and communication.<br><u>Individual determinants</u>:<br>• CWR: physician specialty (internal medicine & pneumology) | | ✓ | ✓ | ✓ |

| Author, year | Main results (Barriers and Facilitators) | Level | | | |
|---|---|---|---|---|---|
| | | System | Organisat. | Inter-indiv. | Individual |
| | • ATC-GP: proximity (as predictor in 1 study and unclear in another), IP experience (contact with pharmacists during formative year), cognitive pharmacy services provision.<br><br>• ATC-P: IP experience (physician contact during training).<br><br>Relational determinants:<br><br>• ATC-GP: expectations, perception of other HCP (role for pharmacist in medication management – indirect predictor), interest for collaborative practice (willingness to collaborate)<br><br>• ATC-P: expectations, perception of other HCP (recognition of roles), interest for collaborative practice (willingness to collaborate)<br><br>• CWR: expectations (practitioner confidence – women), skills (trustworthiness)<br><br>• GPCPC: interest for collaborative practice ("knowing each other")<br><br>Interactional determinants: components of interpersonal relationship among the professionals:<br><br>• ATC-GP: trust, interdependence (mutual respect), communication, role definition (clear roles)<br><br>• ATC-P: trust (indirect predictor), interdependence (mutual respect), communication, role definition (recognition of roles)<br><br>• CWR: interdependence (role specification/ power and justice), communication (trustworthiness), role definition (role specification), systems (professional interaction) | | | | |

| Author, year | Main results (Barriers and Facilitators) | Level | | | |
| --- | --- | --- | --- | --- | --- |
| | | System | Organisat. | Inter-indiv. | Individual |
| Bollen, 2019 | **Barriers**<br><br><u>Negotiating professional boundaries</u>:<br><br>• Lack of experience of professional relationship in collaboration in the past<br><br>• Negative attitudes related to past experiences and other professionals, negative attitudes of mostly older generation GP<br><br>• Attitudes: Distorted perceptions of the role of the other party, difficulties working out role boundaries (e.g. who had the main responsibility for patient therapy)<br><br>• Feelings: CP feeling that GP considered their requests and activity as invading their territory; GP feeling a loss of professional freedom in collaboration, CP feeling undervalued<br><br>• Hierarchy, power: GP perceived CP as retailer, to be subordinate and inferior<br><br>• Trust and respect: some GP showed lack of respect towards CP on the phone, lack of trust and respect<br><br><u>Perceived skills and knowledge</u>:<br><br>• Communication: through third party, lack of communication strategy enabling professionals to deal with changing roles in delivering healthcare services, lack of (face-to-face) communication, frequent telephone consultations of CP, unclear written communication from GP, irregular, inconsistent or reactive communication towards each other, professionals' personal complaints in meetings, challenges in communication when including ethical perspectives during clinical decisions | ✓ | ✓ | ✓ | ✓ |

| Author, year | Main results (Barriers and Facilitators) | Level | | | |
|---|---|---|---|---|---|
| | | System | Organisat. | Inter-indiv. | Individual |
| | • Shared goals (confidence in outcomes): GP and CP negative expectations of collaboration, mostly related to patient care, GP concerns about patient confidentiality (such as patients not wanting to share information with pharmacists, risk to increased fragmented healthcare and duplication of tasks causing increased healthcare costs)<br>• Capabilities: GP perceived CP as having a lack of knowledge and skills regarding (increased) patient care, CP perceptions included that GP lacked knowledge of pharmacists' development of patient care skills and that GP had misconceptions and lacked understanding of pharmacy services<br>• Different perspectives: GP and CP differed in their perceptions of preferred communication methods, extending the pharmacist role (GP seeing them as retailers, while pharmacists perceived themselves as clinically competent) and accessibility to each other's practices<br>• CP contributions<br><u>Structural and organizational facilitators and barriers</u>:<br>• Environment<br>• Lack of time, for both pharmacists and PCP<br>• Lack of remuneration<br>• Inaccessibility, such as difficulties getting access to GP, CP, or patients' medical records, disorganized charting systems in an integrated setting<br>• Management support<br><u>Education, training:</u> | | | | |

| Author, year | Main results (Barriers and Facilitators) | Level | | | |
|---|---|---|---|---|---|
| | | System | Organisat. | Inter-indiv. | Individual |
| | • Education, training: a lack of training and support, a low level of the professionals' educational/training qualification | | | | |
| | **Facilitators**<br><br><u>Negotiating professional boundaries:</u><br><br>• Historical experience with collaboration: previous contact or experience with collaboration, CP full-time presence<br><br>• Attitudes<br><br>• Role specification: understanding of each other's role, mutual recognition<br><br>• Feelings<br><br>• Hierarchy, power<br><br>• Trust and respect: understanding each other's roles, effective communication, a good opinion of the other professional, professionals' commitment to the working relationship, respect towards each other and the willingness to collaborate<br><br><u>Perceived skills and knowledge:</u><br><br>• Regular telephone or face-to-face contact between the two professionals, ensuring CP received feedback from the GP, straightforward, open, honest, regular and proactive communication and information sharing<br><br>• Shared goals (confidence in outcomes): perceived usefulness of other professions, opportunity to take on new professional roles and an easier path to teamwork<br><br>• Capabilities | ✓ | ✓ | ✓ | ✓ |

| Author, year | Main results (Barriers and Facilitators) | Level | | | |
|---|---|---|---|---|---|
| | | System | Organisat. | Inter-indiv. | Individual |
| | • Different perspectives: positive patient perspectives towards delivery of care<br><br>• CP contributions<br><br><u>Structural and organizational facilitators and barriers:</u><br><br>• Environment: co-location, pharmacists being located in more rural areas, use of rules and protocols, presence of a collaboration system, rigorous and improved service business model, availability of sufficient consultation rooms, resources, staff, and communication tools<br><br>• Time<br><br>• Remuneration: additional remuneration<br><br>• Access<br><br>• Management support: management in favor of collaboration, co-operation with professional organizations<br><br><u>Education, training:</u><br><br>• Joint training/education and regular meetings to increase awareness of professionals' skills, person responsible for leading collaboration, stakeholder consultation regarding professional roles, collaboration in integrated settings (in which GP and CP were co-located) facilitated by education sessions and support from mentors | | | | |
| **Intersectoral collaboration** | | | | | |
| Harnagea, 2017 | **Barriers**<br><br><u>Globally:</u> | ✓ | ✓ | ✓ | ✓ |

| Author, year | Main results (Barriers and Facilitators) | Level | | | |
|---|---|---|---|---|---|
| | | System | Organisat. | Inter-indiv. | Individual |
| | • PHP competencies at micro level and in clinical integration.<br>• Low political priority in the system integration domain at macro level<br>• Lack of funds in the organizational integration domain, at meso level.<br>Lack of political leadership and healthcare policies:<br>• Lack of political leadership<br>• Poor understanding of oral health status of population<br>• Low prioritization of oral health on political agenda<br>• Absence of appropriate oral health policies<br>• Insurance policies and separate medical and dental insurance realms<br>• Professional legislation policies (from country) did not allow delivery of preventive oral healthcare by non-dental professionals<br>Implementation challenges:<br>• Cost of integrated services<br>• Human resources issues<br>• Deficient administrative infrastructure<br>• Challenges to ensure economic stability of programs targeting oral health in PC and high cost of equipment maintenance | | | | |

| Author, year | Main results (Barriers and Facilitators) | Level | | | |
|---|---|---|---|---|---|
| | | System | Organisat. | Inter-indiv. | Individual |
| | • Workload of personnel, staff turnover, time constraints and scarcity of various trained human resources such as care coordinators, public health workforce and allied dentists<br><br>• Recruitment and retention of dental and non-dental staff were considered challenging, mostly due to the limited number of professionals interested in working in primary integrated clinics and shortage of dentists in rural and remote regions.<br><br>• deficient administrative infrastructure (such as absence of dental health records in medical records, cross-domain interoperability and domain-specific act codes) considered as contributor to general perception of dental care as an 'optional' service, hindering medical professionals from performing basic dental services.<br><br><u>Discipline-oriented education and lack of competencies:</u><br><br>• Lack of interprofessional education<br><br>• Focusing on discipline-oriented training in health<br><br>• Lack of competencies<br><br>• Knowledge, attitudes and skills were the most reported meaning units of competencies of PHP<br><br>• Lack of knowledge regarding integrated care practices (for both dental and non-dental care providers)<br><br>• Patients and most of PHP did not attribute value to continuity of care in the field of oral health because oral health conditions are rarely life threatening (may be explained by lack of knowledge and awareness of the impact of oral health on general health and well-being) | | | | |

| Author, year | Main results (Barriers and Facilitators) | Level | | | |
|---|---|---|---|---|---|
| | | System | Organisat. | Inter-indiv. | Individual |
| | <u>Patient's oral healthcare needs:</u><br>• Patients' decision to accept or refuse integrative care was mainly based on their need perception rather than the assessment of healthcare providers | | | | |
| | **Facilitators**<br><u>Globally</u>:<br>• Collaborative practices in the functional domain and financial support in the system integration domain, at the macro level<br><u>Supportive policies and resources allocation:</u><br>• Importance of financial support from governments, stakeholders and non-profit organizations at the macro level<br>• Partnerships and common vision among governments, communities, academia, various stakeholders and non-profit organizations<br>• Healthcare policies and reimbursements to trained PCP for oral screening, patient education and fluoride varnish applications<br><u>Interprofessional education and PCP in preventive oral health</u><br><u>Collaborative practices</u>:<br>• Perceived responsibility and role identification, case management and incremental approach.<br><u>Local strategic leaders:</u> | ✓ | ✓ | ✓ | |

| Author, year | Main results (Barriers and Facilitators) | Level | | | |
| --- | --- | --- | --- | --- | --- |
| | | System | Organisat. | Inter-indiv. | Individual |
| | • Strategic role of local leader in building teamwork and communities' capacities (registered nurses or oral health coordinator)<br><br><u>Proximity:</u><br><br>• Geographical proximity or colocation of dental and medical practices | | | | |
| Leenaars, 2015 | **Barriers**<br><br><u>Collaborative initiatives to refer PC patients to sport facilities</u><br><br>• Lack of communication<br><br>• Lack of feedback from sport or leisure professionals on patients' progress<br><br>• Leisure or sport professionals' limited medical knowledge (physicians and physiotherapists 'unsure' and 'apprehensive' of PA program for patients and 'uncomfortable' with leisure or fitness professionals).<br><br>• Physicians' and physiotherapists' lack of time caused problems for physicians to implement interventions<br><br>• High number of patient referrals led to overcrowding, resulting in reduced number of referred patients who could use the facility<br><br><u>Collaborative initiatives to organize activities to promote PA among the community</u><br><br>• Lack of communication<br><br>• Unclear roles and responsibilities<br><br>• Staff turnover in professional organizations<br><br>• Lack of agency capacity | ✓ | ✓ | ✓ | ✓ |

| Author, year | Main results (Barriers and Facilitators) | Level | | | |
|---|---|---|---|---|---|
| | | System | Organisat. | Inter-indiv. | Individual |
| | • Lack of leadership<br>• Uncertainty about funding<br>• Differences between shared interest of professional organizations (interest in the program) and of volunteer groups (increased club memberships), different cultures in PC and sport sector (preferred meeting time and target groups) led to difficulties in engaging sport organizations in the partnership<br>• Sport organizations did not always recognize benefits of partnership or were not familiar with types of participant in intervention program (obese people, often in combination with low socio-economic status)<br>• Health professionals' lack of time to establish partnerships or to refer patients | | | | |
| | **Facilitators**<br><u>Collaborative initiatives to refer PC patients to sport facilities</u><br>• Collaboration provided physicians with a better understanding and awareness of the services and support available to their patients<br>• Referral scheme also laid groundwork for a relationship between physicians and sport organizations<br>• Referral process provided a welfarist and commercial benefit for leisure<br>• Funding or remuneration as a priority or a key influence on ongoing implementation<br><u>Collaborative initiatives to organize activities to promote PA among the community</u><br>*For partnership formation:* | ✓ | ✓ | ✓ | ✓ |

| Author, year | Main results (Barriers and Facilitators) | Level | | | |
|---|---|---|---|---|---|
| | | System | Organisat. | Inter-indiv. | Individual |
| | • Trust and shared interests among members<br>• Having enough time to develop trust among members<br>• Funding<br>• Formalization of partnership<br>• Agreement<br>• Engagement of key stakeholders<br>*For the partnership function:*<br>• Short communication lines and communicating roles and responsibilities<br>• Organization's capacity to participate and develop programs<br>• Organization's commitment<br>• Engagement of more than one person from a sport organization (key leaders that influence strategic direction of the sports club)<br>• Professional organization (reduce impact of staff turnover)<br>• Visibility of results for the partners | | | | |
| Kirst, 2017 | **Barriers** *(identified in mixed results and unsuccessful program)*<br>• Challenges to provider commitment: lack of incentives for physicians to participate in IC program activities, limited provider enthusiasm from significant changes in practice (from implementation), and limited flexibility for providers to make operational changes; providers viewed team meetings as time-consuming, and did not fully understand how to use program protocols due to limited training ; GP under a | | ✓ | ✓ | ✓ |

| Author, year | Main results (Barriers and Facilitators) | Level | | | |
|---|---|---|---|---|---|
| | | System | Organisat. | Inter-indiv. | Individual |
| | fee-for-service model less likely to become engaged and commit to the model due to uncompensated time for multidisciplinary team meetings and other program activities<br><br>• Challenges to team collaboration: short trial period (12 months) for providers to build trusting relationships within multidisciplinary teams | | | | |
| | **Facilitators** *(identified in successful programs)*<br><br><u>Trusting multidisciplinary team relationships</u><br><br>• Quality of multidisciplinary team relationships (mechanism) to effective team collaboration and success of IC programs<br><br>• Cross-sector multidisciplinary teams spanning different organizations, trusting each other, with clear roles, and relying on each other to perform respective roles, collaborated closely and communicated effectively, shared knowledge about their work and patient information more effectively, which allowed for continuity of care and better coordination of care<br><br>• Strong leadership to guide teams helped to build trust and support team collaboration<br><br>• Leadership promoting organizational culture fostering a shared vision of IC programs, involved joint ownership and accountability across partnering organizations, supported trust building and collaborative team functioning<br><br>• Developing a shared vision supported by leadership for trust building<br><br>• Creating a culture of team participation supported by leadership to generate a sense of ownership and strengthen relationships among team members, to make staff feel comfortable with model, encouraging them to take an active role in team work | ✓ | ✓ | ✓ | ✓ |

| Author, year | Main results (Barriers and Facilitators) | Level | | | |
|---|---|---|---|---|---|
| | | **System** | **Organisat.** | **Inter-indiv.** | **Individual** |
| | • Open team communication supported timely transfers of information between team members, in making informed, collaborative decisions regarding patient care<br><br>• In some programs, team co-location and/or clinical champions promoting IC model facilitated relationship building, contributing to teams collaborating more effectively<br><br>• Provision by leadership of time and support for teams to build trusting relationships facilitated collaboration and communication<br><br>Provider commitment to and understanding of the program model<br><br>• Provider understanding, commitment and belief in the model to motivate providers to make the effort to change their work practices and work multi-disciplinarily<br><br>• Providers having an understanding of the process of care under the model, their roles in this process, and benefits of the model for patient care (for implementation)<br><br>• Contextual factors facilitating provider commitment and understanding included funding models that involved incentives for providers to implement IC<br><br>• More flexibility and resources for GP in capitated programs and programs with salaried staff<br><br>• Expertise of providers for building an understanding of the model and team work<br><br>• Investment in providers' ongoing training and how to work together effectively in teams to build provider expertise<br><br>• Establishment of an organizational culture involving a shared vision fostered by strong leadership, increased provider understanding of the model and subsequent collaborative team functioning, and providing guidance in implementation | | | | |

| Author, year | Main results (Barriers and Facilitators) | Level | | | |
|---|---|---|---|---|---|
| | | System | Organisat. | Inter-indiv. | Individual |
| | • Time to set up infrastructure for program implementation, involving building team relationships, establishing coordination across partnering organizations and information management systems, enrolling patients and developing appropriate care plans<br>• Flexibility in implementation supported provider commitment to the model<br>• Programs adapted to population's local needs by allowing operational changes, and aligning care with population needs over time served to build provider commitment, enthusiasm and confidence in implementing the model and made providers, especially GP, feel that it was an effective program for meeting patients' needs | | | | |
| Davies, 2011 | **Barriers** *(to integrated working)*<br>• NHS staff: failure to acknowledge expertise, difficulty of gaining trust and cynicism towards care home expertise and staff<br>• Lack of access to NHS services<br>• High staff turnover and lack of access to training<br>• Lack of staff knowledge and confidence<br>• Care homes professionally isolated<br>• Lack of teamwork in care homes | | ✓ | ✓ | |
| | **Facilitators** *(to integrated working)*<br>• Care homes valued NHS input and training<br>• 'Bottom-up' approach to train staff so that all levels of staff are involved | ✓ | ✓ | ✓ | |

| Author, year | Main results (Barriers and Facilitators) | Level | | | |
|---|---|---|---|---|---|
| | | System | Organisat. | Inter-indiv. | Individual |
| | • Health care professionals acting as advocates for care homes in relation to care, sharing good practice and enabling care home staff to network and promoting better access to services for the care home<br>• Care home managers supporting the intervention and ensuring access to training for staff, through establishing learning contracts<br>• Protected time, inclusion of all levels of care home staff for training and support by health care professionals | | | | |
| Martin-Misener, 2012 | **Facilitators**<br><u>Systemic level factors</u><br>• Government involvement<br>• Policy and fit with local needs<br>• Funding and resource factors<br>• Power and control issues<br>• Education and training<br><u>Organizational factors</u><br>• Necessity of a common agenda<br>• Knowledge and resource (human and financial resources, space, team building and change management capacity)<br>• Leadership, management and accountability issues<br>• Partners' geographic proximity | ✓ | ✓ | ✓ | ✓ |

| Author, year | Main results (Barriers and Facilitators) | Level | | | |
|---|---|---|---|---|---|
| | | System | Organisat. | Inter-indiv. | Individual |
| | • Shared protocols, tools and information sharing<br>Interactional factors<br>• Shared purpose; philosophy and beliefs<br>• Clear roles and positive relationships<br>• Effective communication and decision-making strategies | | | | |

[a]ATC-GP: general practitioners' attitudes towards collaboration with community pharmacists; ATC-P: community pharmacists' attitudes towards collaboration with general practitioners' model; CC: Collaborative care; CM: Care managers; CP: community pharmacists CPS: Community pharmacy services; CWR: Collaborative Working Relationship Model; EMR: Electronic medical records; GP: General practitioners; GPCPC: General Practitioner and Community Pharmacist collaboration; HCP: Health care providers; HIPAA: Health Insurance Portability and Accountability Act; IC: Integrated care; IP: Interprofessional experience; IPCT: Interprofessional primary care teams; IT: Information technology; MPC: Multi-professional collaboration; NHS: National Health Service; NPT: Normalization process theory; NP: Nurse practitioners; PA: Physical activity; PC: Primary care; PCT: Primary care teams; PHP: Primary healthcare providers
